# Simultaneous *T*_2_, *T*_2_*, and *R*_2_′ Mapping for Multiple Sclerosis Using Nonlinear Model‐Based Reconstruction of Undersampled Radial RARE‐EPI MRI

**DOI:** 10.1002/mrm.70465

**Published:** 2026-06-18

**Authors:** Jose Raul Velasquez Vides, Carl J. J. Herrmann, Thomas Gladytz, Hoby P. Hetherington, Hendrik Mattern, Xiaoqing Wang, Jason M. Millward, Shahriar Shalikar, Igor Fabian Tellez Ceja, Beate Endemann, Sonia Waiczies, Joseph Kuchling, Friedemann Paul, Georg Rose, Min‐Chi Ku, Franz Schmitt, Thoralf Niendorf

**Affiliations:** ^1^ Max‐Delbrück‐Center for Molecular Medicine in the Helmholtz Association (MDC), Berlin Ultrahigh Field Facility (B.U.F.F.) Berlin Germany; ^2^ Institute for Medical Engineering, Otto‐von‐Guericke University Magdeburg Germany; ^3^ Department of Physics Humboldt Universität zu Berlin Berlin Germany; ^4^ Resonance Research Inc. Billerica Massachusetts USA; ^5^ Department of Biomedical Magnetic Resonance Otto‐von‐Guericke University Magdeburg Germany; ^6^ German Center for Neurodegenerative Diseases (DZNE) Magdeburg Germany; ^7^ Center for Behavioral Brain Sciences (CBBS) Magdeburg Germany; ^8^ Department of Radiology Boston Children's Hospital, Harvard Medical School Boston Massachusetts USA; ^9^ Experimental and Clinical Research Center (ECRC), a Joint Cooperation Between the Charité Medical Faculty and the Max‐Delbrück‐Center for Molecular Medicine in the Helmholtz Association Berlin Germany; ^10^ Charité – Universitätsmedizin Berlin Berlin Germany; ^11^ Department of Neurology Charité – Universitätsmedizin Berlin Berlin Germany; ^12^ NeuroCure Clinical Research Center, Charité – Universitätsmedizin Berlin Berlin Germany; ^13^ Research Campus STIMULATE, Otto‐von‐Guericke University Magdeburg Germany

**Keywords:** central vein sign, multiple sclerosis, nonlinear model‐based reconstruction, quantitative multiparametric MRI, *R*_2_′ mapping, simultaneous *T*_2_ and *T*_2_* mapping

## Abstract

**Purpose:**

To demonstrate the synergy of undersampled radial 2in1‐RARE‐EPI acquisition and nonlinear model‐based reconstruction for accelerated and simultaneous *T*_2_, *T*_2_*, and *R*_2_′ mapping in brains of patients with multiple sclerosis (MS).

**Methods:**

2in1‐RARE‐EPI combines a RARE module with an EPI module to capture *T*_2_ and *T*_2_* information. Nonlinear model‐based reconstruction was applied to estimate *T*_2_, *T*_2_* maps directly from undersampled *k*‐space data. A retrospective undersampling experiment was conducted to compare nonlinear model‐based and parallel imaging compressed sensing (PICS) reconstruction. The proposed approach was validated and compared to reference methods multiecho spin‐echo (*T*_2_, MSE) and multiecho gradient‐echo (*T*_2_*, MGRE) in a phantom, healthy subjects, and MS patients.

**Results:**

2in1‐RARE‐EPI together with nonlinear model‐based reconstruction enabled *T*_2_, *T*_2_*, and *R*_2_′ mapping with 7.5‐fold scan‐time acceleration relative to the references, while addressing key limitations of reference techniques, including long acquisition times, misregistration, motion and off‐resonance sensitivity, and the need for calibration scans. Phantom and in vivo validation showed that the parametric maps obtained with this approach were in agreement with the reference methods. Compared with PICS, nonlinear model‐based reconstruction showed more consistent spatial detail and accuracy at higher acceleration factors. The proposed method detected small focal lesions in *T*_2_, *T*_2_*, and *R*_2_′ maps of MS patients and enabled visualization of the central vein sign.

**Conclusion:**

Scan time reduction facilitated by nonlinear model‐based reconstruction of 2in1‐RARE‐EPI provides a technical foundation for enhanced patient compliance, and is a fundamental precursor for broader clinical studies on the potential of *T*_2_, *T*_2_*, and *R*_2_′ as imaging biomarkers.

## 1 Introduction

Quantitative magnetic resonance imaging (qMRI) provides enhanced sensitivity to brain tissue alterations associated with neurodegenerative and neuroinflammatory diseases such as multiple sclerosis (MS) [1, 2, 3, 4, 5]. MR relaxometry is particularly useful for assessing MS beyond lesion detection. *T*_2_ prolongation in normal‐appearing white matter (NAWM) of MS patients has been linked to disease severity [6, 7, 8]. *T*_2_* mapping has been used to assess iron accumulation, underlying pathological mechanisms in MS [9, 10, 11], and *R*_2_′ provides higher specificity for iron accumulation [9, 12, 13, 14].

$T_{2}\;\text{and}\;T_{2}^{*}$ mapping techniques use multiecho spin‐echo (MSE) and multiecho gradient‐echo (MGRE) acquisitions, respectively, but are limited by long acquisition times, sensitivity to motion and off‐resonance effects, and spatial misregistration. To address these challenges, recent developments have focused on simultaneous $T_{2}\;\text{and}\;T_{2}^{*}$ acquisitions. These include GRASE [15] variants [16, 17, 18, 19, 20, 21, 22], single‐shot acquisitions based on spatiotemporal encoding [23, 24], MR fingerprinting [25], and *T*_2_‐prepared GRE readouts [26, 27, 28, 29]. Among these approaches, sensitivity to the underlying relaxation parameters is a key performance criterion. The 2in1‐RARE‐EPI technique [30, 31, 32, 33] fulfills this requirement by combining a RARE module with an EPI module, enabling separate encoding of $T_{2}\;\text{and}\;T_{2}^{*}$ decay information. Consequently, $T_{2}\;\text{and}\;T_{2}^{*}$ maps of 2in1‐RARE‐EPI data can be reconstructed independently, using the most appropriate reconstruction method. However, the choice of reconstruction strategy plays an important role in the quality and reliability of parameter estimation from undersampled data.

Typically, qMRI reconstruction involves reconstructing multicontrast images followed by voxel‐wise fitting for parameter estimation. For image reconstruction, regularized techniques such as compressed sensing (CS) [34] and low‐rank reconstructions [35] leverage intrinsic prior knowledge of image sparsity and the low‐rank structure of multicontrast data. Subspace‐constrained reconstruction models tissue signal evolutions within a linear subspace, enabling reconstruction of only a small set of subspace coefficient images [36, 37]. For parameter estimation, dictionary matching is commonly used [20, 26, 38, 39, 40], where expected signal evolutions are generated using Bloch simulations or extended phase graph (EPG) modeling [41]. An efficient alternative to this two‐step workflow is nonlinear model‐based reconstruction [42, 43, 44, 45, 46, 47], which directly estimates quantitative parameters from undersampled *k*‐space data using iterative optimization, incorporating the MR signal model as explicit prior information in the reconstruction.

We previously explored the feasibility of accelerated simultaneous $T_{2}\;\text{and}\;T_{2}^{*}$ mapping using 2in1‐RARE‐EPI with CS reconstruction [48]. However, this initial approach suffered from loss of spatial resolution at higher acceleration factors, increased sensitivity to off‐resonances, and it required a prescan for gradient delay correction. These limitations prevented reliable calculation of *R*_2_′ maps (*R*_2_′ = 1/*T*_2_* − 1/*T*_2_ = *R*_2_* − *R*_2_).

To overcome these limitations, we propose a synergistic combination of 2in1‐RARE‐EPI with nonlinear model‐based reconstruction, which is conceptually appealing for several reasons: (1) nonlinear model‐based reconstructions benefit from acquisition strategies with simple analytical signal models, since the signal model is embedded in the forward operator whose derivatives are required when evaluating the optimization cost function. In 2in1‐RARE‐EPI, $T_{2}\;\text{and}\;T_{2}^{*}$ relaxations follow two independent exponential models that are easy to differentiate. (2) Nonlinear problems are dependent on parameter initialization [49]. Here, the RARE module is an easier problem to solve because it is less affected by off‐resonance modulation and provides suitable initial maps for solving the EPI problem, for example, by using the *T*_2_ map as an initial *T*_2_* estimate. (3) Nonlinear model‐based formulations allow the incorporation of sparsity and smoothness constraints directly on the parameter maps [46], improving robustness to noise and undersampling. (4) Nonlinear model‐based reconstructions estimate parameter maps together with *B*_0_ and coil sensitivity maps within the same optimization framework, thereby potentially reducing sensitivity to prescan miscalibrations (e.g., due to subject motion) and eliminating the additional acquisition time associated with separate calibration scans. (5) Radial *k*‐space sampling in 2in1‐RARE‐EPI promotes incoherent undersampling and improves robustness to motion [50], which generally improves regularized nonlinear reconstruction [43]. Based on these considerations, the proposed approach would yield $T_{2},\;T_{2}^{*},\text{and}\;R_{2}^{\prime }$ maps with improved preservation of spatial detail at high acceleration factors, reduced sensitivity to off‐resonance effects and sensitivity map imperfections, and improved depiction of MS lesions and their internal structure.

This study demonstrates the feasibility of combining undersampled 2in1‐RARE‐EPI with nonlinear model‐based reconstruction for accelerated, simultaneous $T_{2},\;T_{2}^{*},\;\text{and}\;R_{2}^{\prime }$ mapping in a phantom, healthy subjects, and MS patients. Reduced scan time facilitated by this approach fosters patient comfort, and may support the clinical translation of $T_{2},\;T_{2}^{*},\;\text{and}\;R_{2}^{\prime }$ as imaging biomarkers in MS, including drug trials, prognostic evaluations, therapeutic decision‐making and monitoring of clinical outcomes [5, 51, 52, 53].

## 2 Methods

### 2.1 2in1‐RARE‐EPI

**FIGURE 1:**
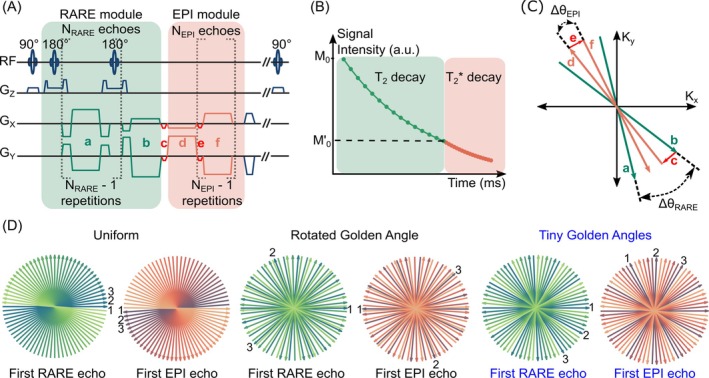
Basic scheme of 2in1‐RARE‐EPI and implemented *k*‐space trajectories. (A) Pulse sequence diagram of 2in1‐RARE‐EPI. (B) Ideal signal evolution. (C) *k*‐Space representation showing the transition between the last two RARE spokes (a, b, separated by $∆\theta_{\text{RARE}}$) and the first two EPI spokes (d, f, separated by $∆\theta_{\mathrm{EPI}}$). (D) *k*‐Space trajectories of the first echo for the RARE and EPI modules under three radial sampling schemes: uniform, rotated golden angle, and tiny golden angles. The spokes are color‐coded indicating their chronological acquisition (intense to light color). The first three spokes are labeled in each *k*‐space trajectory.

2in1‐RARE‐EPI combines a RARE [54] module with an EPI [55] module (Figure 1A) to capture $T_{2}\;\text{and}\;T_{2}^{*}$ decay information (Figure 1B) [56, 57, 58]. The RARE module fulfills the Carr–Purcell–Meiboom–Gill (CPMG) [59, 60, 61] condition. Crusher gradients are applied along the slice‐selection direction, each introducing an intravoxel dephasing of 4*π* [59, 62]. The transition between the last RARE spoke and the first EPI spoke, and between subsequent EPI spokes, is achieved using small blip gradients (Figure 1C). The spoiler gradient is oriented in the same direction as the final EPI readout gradient to obtain more homogeneous spoiling [63], giving a total phase dispersion of 4*π* [59, 64, 65].

### 2.2 *k*‐Space Trajectory

We implemented a tiny golden angle radial sampling scheme [66] designed to meet four criteria: (i) sufficient spacing in *k*‐space between consecutively acquired spokes to minimize clustering of motion‐induced errors [67, 68], (ii) a nearly uniform distribution of spokes in *k*‐space for a given echo to reduce undersampling artifacts, (iii) opposing orientations of adjacent spokes for a given echo to reduce off‐resonance artifacts [42, 69], and (iv) the last RARE echo providing complementary information to the EPI echoes for *T*_2_* mapping. The RARE and EPI modules used angle increments of $∆\theta_{\text{RARE}}\approx 23.6{}^{\circ}\;\text{and}∆\theta_{\mathrm{EPI}}\approx 10.2{}^{\circ}$. For comparison, uniform and rotated golden angle schemes [70] satisfying criteria (ii) and (iv) only, were also implemented. A description of the sampling schemes is provided in the Supporting Information S1. Figure 1D shows *k*‐space trajectories of the first echo in both modules for the three schemes, using echo train lengths ${\mathrm{ETL}}_{\text{RARE}}=14,{\mathrm{ETL}}_{\mathrm{EPI}}=18$, and 37 RF excitations.

### 2.3 Nonlinear Signal Models

For multicoil, multiecho experiments, the forward discrete MR signal model can be written as [36, 43, 44, 45]:

(1) $$y_{j,n}={\vec{F}}_{j,n}\left(x\right)≔P_{n}ℱCM_{n},$$

where $j\in 1,\ldots ,N_{\text{coil}}$ is the coil index, and n∈1,…,ETL is the echo index. ${\vec{F}}_{j,n}\left(x\right)$ represents the nonlinear forward operator that maps the unknown parameters x to the measured data y. $P_{n}$ is the projection onto the *k*‐space trajectory, ℱ the Fourier transform, C the operator that multiplies the coil sensitivities $c_{j}$ with the echo images, and $M_{n}$ the nonlinear signal model.

For the RARE module, the unknown parameters are $x={\left(M_{0},R_{2},\ldots ,c_{j},\ldots \right)}^{T}$, and $M_{n}$ is modeled as:

(2) $$M_{n}\left(M_{0},R_{2},\vec{r}\right)=M_{0}\left(\vec{r}\right)\cdot e^{-R_{2}\left(\vec{r}\right)\cdot {\mathrm{TE}}_{n}},$$

where $\vec{r}$ denotes the spatial position in image space, $M_{0}$ the spin density map, $R_{2}$ the transverse relaxation rate, and ${\mathrm{TE}}_{n}$ the nth RARE echo time. For the EPI module, the unknowns are $x={\left(M_{0}^{\prime },R_{2}^{*},f_{B_{0}},\ldots ,c_{j},\ldots \right)}^{T}$, and $M_{n}$ is modeled as:

(3) $$M_{n}\left(M_{0}^{\prime },R_{2}^{*},f_{B_{0}},\vec{r}\right)=M_{0}^{\prime }\left(\vec{r}\right)\cdot e^{-R_{2}^{*}\left(\vec{r}\right)\cdot {\mathrm{TE}}_{n}^{\prime }}\cdot e^{i2\pi \cdot f_{B_{0}}\left(\vec{r}\right)\cdot {\mathrm{TE}}_{n}^{\prime }},$$

where $R_{2}^{*}$ is the effective transverse relaxation rate, ${\mathrm{TE}}_{n}^{\prime }$ the nth EPI echo time, $f_{B_{0}}$ the *B*_0_ field inhomogeneity map, and $M_{0}^{\prime }\left(\vec{r}\right)=M_{0}\left(\vec{r}\right)\cdot e^{-R_{2}\left(\vec{r}\right)\cdot {\mathrm{TE}}_{\text{lastRARE}}}$ the remaining transverse magnetization after the last RARE echo.

### 2.4 Nonlinear Model‐Based Reconstruction

We solved two nonlinear inverse problems for data obtained from the RARE and EPI modules of 2in1‐RARE‐EPI, respectively, of the form:

(4) $$\hat{x}=\underset{x}{\text{argmin}}\;\sum_{n}\;\sum_{j}{\left\|F_{j,n}\left(x\right)-y_{j,n}\right\|}_{2}^{2}+\sum_{i}\lambda_{i}R_{i}\left(x\right)$$

The first term is the data fidelity term, and the second term consists of regularizers $R_{i}$ controlled by the regularization parameters $\lambda_{i}$.

BART (version 0.7.00) [71] was used for offline nonlinear model‐based reconstruction. The multicoil *k*‐space data were noise prewhitened [72] using the scanner‐provided noise prescan, from which the coil noise covariance matrix was estimated and its Cholesky factor used for decorrelation [73]. To reduce computational load, coil compression via principal component analysis [74, 75] was applied, reducing the receive coil number from 32 to 12. The *k*‐space trajectory was corrected for gradient delay errors using the radial spoke intersections for gradient delay estimation (RING) method [76], which is based on the ellipse model of gradient delays [77, 78]. RING estimates the trajectory shifts from spoke intersections to correct the nominal trajectory before reconstruction.

For the RARE problem, a joint $\ell_{1}$‐Wavelet regularization [46, 79] with $\lambda_{1}$ was applied to ${\left(M_{0},R_{2}\right)}^{T}$ to control noise. To enforce spatial smoothness of the coil sensitivity maps ${\left(\ldots ,c_{j},\ldots \right)}^{T}$, we used Sobolev regularization [80] defined as $R\left(\cdot \right)={\left\|{\left(1+s{\left\|\vec{k}\right\|}^{2}\right)}^{l/2}\;ℱ\left\{\cdot \right\}\right\|}^{2}$, where $\left\|\vec{k}\right\|$ defines the distance to the *k*‐space center. The Sobolev parameters were set to s=220andl=15. Equation (4) was solved by an iteratively regularized Gauss–Newton method (IRGNM) [81] using m=10 Newton iterations. In each Newton step, Equation (4) is linearized and the resulting linear subproblem is solved by the fast iterative shrinkage‐thresholding algorithm (FISTA) [46, 82]. During each Newton iteration the number of FISTA iterations was increased according to $N_{m}=\min\left(250,2\cdot N_{m-1}\right)$ with $N_{0}=10$. All parameter ${\left(M_{0},R_{2},\ldots ,c_{j},\ldots \right)}^{T}$ were initialized to one. The first RARE echo was not included in the reconstruction to mitigate *T*_2_ overestimation due to stimulated echoes [83, 84].

For the EPI problem, joint $\ell_{1}$‐Wavelet regularization with $\lambda_{2}$ as well as total variation (TV) [85] with $\lambda_{3}$ were applied to ${\left(M_{0}^{\prime },R_{2}^{*}\right)}^{T}$, and $\ell_{2}$ regularization with $\lambda_{4}$ was applied to all unknowns. Sobolev regularization was applied to the coil sensitivities (s=220,l=15) and $f_{B_{0}}$ (s=5,l=0.5) maps. Equation (4) was solved using the IRGNM algorithm using m=15 Newton iterations and the resulting linear subproblems were solved using the alternating direction method of multipliers (ADMM) [86]. During each Newton iteration the number of ADMM iterations was increased according to $N_{m}=\min\;\left(200,10\cdot 2^{-\ln\lambda^{\left(m\right)}}\right)$. For this problem, the off‐resonance phase modulation term introduces multiple local minima in the cost function. Reasonable initial parameter maps, therefore, help the reconstruction converge to a suitable solution [42, 45, 49, 87]. We first solved the RARE problem to obtain ${\left(M_{0},R_{2}\right)}^{T}$, and then generated a synthetic [88] last RARE echo image, which was used as the initial magnetization $M_{0}^{\prime }$. The $R_{2}$ map was scaled and used as the initial $R_{2}^{*}$ estimate, with the scaling factor set to 2 (1.4 for phantom data), based on the prior knowledge that $R_{2}^{*}>R_{2}$. An initial $f_{B_{0}}$ map was obtained from the first three EPI echoes [87]. Coil sensitivities were initialized to zero. The last RARE echo was included as the first EPI echo in the reconstruction.

For both reconstructions, the four regularization parameters were initially set to $\lambda_{i}^{0}=1$, and then reduced along Newton steps according to $\lambda_{i}^{\left(m\right)}=\max\left(\lambda_{i}^{0}\cdot {\left(1/G\right)}^{m-1},\;\lambda_{\min}\right)$ where m is the mth Newton step and G a reduction factor, set to 2 and 2.5 for the RARE and EPI problem, respectively. The minimum regularization values $\lambda_{\min}$ were chosen to suppress noise while preserving image details, set to $\lambda_{\min}=0.005$ for the RARE problem and $\lambda_{\min}=0.0002$ for the three regularizers in the EPI problem. A grid search was performed to select the regularization parameter values with the final choice justified by visual inspection (Figures S1–S3).

The nonlinear model‐based reconstruction was executed on a GPU (NVIDIA GeForce RTX 3090, 24 GB memory), with a computation time of 65 s per slice for *T*_2_ mapping and 67 s per slice for *T*_2_* mapping.

Vendor‐provided 2D Cartesian MSE and MGRE sequences were used as reference for $T_{2}\;\text{and}\;T_{2}^{*}$ mapping, respectively, applying prewhitening and coil compression. For multislice MR acquisitions, an interleaved acquisition scheme was used for 2in1‐RARE‐EPI and MSE, and a sequential slice acquisition was used for MGRE. For MSE and MGRE, Echo images were reconstructed with the CG‐SENSE [89] algorithm implemented in BART, with coil sensitivities estimated from the first RARE echo using ESPIRiT [90]. MSE‐*T*_2_ maps were generated via voxel‐wise magnitude fitting to the model in Equation (2). MGRE‐*T*_2_* maps were obtained analogously using Equation (3), omitting the off‐resonance phase modulation term. Fitting was performed using a nonlinear least‐squares IRGNM implementation in BART. Complex fitting of the MGRE data becomes unstable in regions with steep susceptibility gradients (Figure S4), motivating the use of magnitude fitting for the MGRE‐*T*_2_* maps.

For the 2in1‐RARE‐EPI data and the reference methods, *R*_2_′ maps were calculated as *R*_2_′ = 1/*T*_2_*‐1/*T*_2_ = *R*_2_*‐*R*_2_. Negative voxel values in the *R*_2_* maps (in air‐filled sinuses and in areas affected by flow artifacts in CSF) derived from both 2in1‐RARE‐EPI and the MGRE reference were set to an arbitrary value of 0.5 s^−1^ to enforce physical plausibility [91]. This value was chosen instead of zero to avoid infinite *T*_2_* values, yielding a relaxation time of 2 s. Navia (*T*_2_) and Lipari ($T_{2}^{*},R_{2}^{\prime }$) colormaps [92] were used for visualization.

### 2.5 MR Study

All measurements were performed on a SkyraFit 3T system (Siemens Healthineers, Erlangen, Germany) using a 32‐channel head RF coil (Siemens Healthineers) for signal reception and the body RF coil for RF transmission.

### 2.6 *B*_0_ Shimming

Achieving a uniform *B*_0_ field is crucial for *T*_2_* mapping, as static magnetic field inhomogeneities cause *T*_2_* shortening that overshadows subtle *T*_2_* changes due to brain tissue pathology [93]. Clinically, *B*_0_ shimming is achieved using the built‐in hardware and software provided by the MRI manufacturers. Here we used and evaluated a software‐based external *B*_0_ shimming tool (Neuroshim Pro V2, Resonance Research Inc., Billerica MA, USA) for enhancing *B*_0_ uniformity. It uses the BOLERO RF pulse sequence [94] and the logical temporal unwrapped shimming (LOTUS) method [95] for calculating the *B*_0_ map and shim currents. The BOLERO acquisition parameters were: TE = 3, 7, 21 ms, TR = 473 ms, flip angle = 20°, FOV = 216 × 216 mm^2^, matrix size = 72 × 72, slice number = 21; slice thickness = 3 mm, distance factor = 100%; receiver bandwidth/pixel = 1006 Hz/pixel. LOTUS shimming was performed prior to all phantom and human measurements.

### 2.7 Phantom Validation

We used a phantom with eight vials containing agar concentrations of 0.5%–5%, all doped with 1 mM of NiSO_4_. It also included one vial with oil, one with distilled water, and two empty vials, to introduce susceptibility and chemical shift effects. For accelerated 2in1‐RARE‐EPI experiments, the acquisition parameters were: FOV = 256 × 256 mm^2^, matrix size = 256 × 256, twofold readout oversampling, TR = 2000 ms, receiver bandwidth = 810 Hz/pixel, ${\mathrm{ETL}}_{\text{RARE}}/{\mathrm{ETL}}_{\mathrm{EPI}}=$ 14/18, echo spacings $\;{\mathrm{ESP}}_{\text{RARE}}/{\mathrm{ESP}}_{\mathrm{EPI}}$ = 6.68/2.46 ms (ESP from last RARE echo to first EPI echo = 2.42 ms), slice thickness = 5 mm, one slice. The number of RF excitations was set to $N_{\text{shot}}=38$, corresponding to an acceleration factor of $R_{\mathrm{Nyq}}=402/38=10.5$, where $R_{\mathrm{Nyq}}$ denotes acceleration relative to the Nyquist number of spokes for a fully sampled acquisition. The acquisition time was TA = 1:16 min:s. Reference cartesian MSE and MGRE acquisitions were acquired with geometries and timings identical to 2in1‐RARE‐EPI, but with ${\mathrm{ETL}}_{\mathrm{MSE}}/{\mathrm{ETL}}_{\text{MGRE}}=14/12$, RF excitations = 256, ${\mathrm{ESP}}_{\mathrm{MSE}}/{\mathrm{ESP}}_{\mathrm{MSE}}=8.6/2.46\;\mathrm{ms}$, ${\mathrm{TR}}_{\mathrm{MSE}}/{\mathrm{TR}}_{\text{MGRE}}=2000/50\;\mathrm{ms}$, ${\mathrm{TA}}_{\mathrm{MSE}}/{\mathrm{TA}}_{\text{MGRE}}$ = 8:36/0:14 min:s, MGRE flip angle = 12°.

### 2.8 Ethics Statement

This study was approved by the local ethics committee (Charité – Universitätsmedizin Berlin, Berlin, Germany, EA1/191/19). Informed written consent was obtained from each participant.

### 2.9 Human Study

Human studies were performed to demonstrate the clinical feasibility of the proposed approach. We recruited 10 healthy volunteers (sex: female (*n* = 4)/male (*n* = 6); age: 26–60 years) and eight patients with MS fulfilling the 2024 McDonald criteria [96] (sex: female (*n* = 2)/male (*n* = 6); age: 26–62 years).

We evaluated the undersampling capabilities of the proposed approach and compared it with parallel imaging and compressed sensing (PICS) reconstruction. A 2in1‐RARE‐EPI brain data set was acquired from one volunteer using the same acquisition parameters as in the phantom study, but with slice thickness = 3 mm and 256 RF excitations $\left(R_{\mathrm{Nyq}}=1.57\right)$. Echo images were then reconstructed using CG‐SENSE [89], with coil sensitivities estimated from the first RARE echo using ESPIRiT. The *T*_2_ map was generated via voxel‐wise magnitude fitting to the model in Equation (2), and the *T*_2_* map was obtained analogously using Equation (3), omitting the off‐resonance phase modulation term. Fitting was performed using a nonlinear least‐squares IRGNM implementation in BART. These served as reference maps. The data set was then retrospectively undersampled with acceleration factors $R_{\mathrm{Nyq}}=10.5,13,16,25\;\text{and}\;33$, and reconstructed using the proposed nonlinear model‐based approach, as well as PICS reconstruction employing joint $\ell_{1}$‐Wavelet regularization across echoes and TV regularization along the echo dimension. $T_{2}\;\text{and}\;T_{2}^{*}$ maps for PICS reconstructions were computed using the same voxel‐wise magnitude fitting procedure as for the CG‐SENSE reference.

For validating our approach against the reference methods, eight subjects and eight MS patients were included. We used the same protocol as in the phantom study (2in1‐RARE‐EPI, MSE, MGRE) with identical acquisition parameters but with slice number = 7, slice thickness = 3 mm with no slice gaps [97], ${\mathrm{TA}}_{2\text{in}1}/{\mathrm{TA}}_{\mathrm{MSE}}/{\mathrm{TA}}_{\text{MGRE}}=1:16/8:36/1:30\;\min:s$. Only seven slices were acquired due to the high RF power deposition requirements for the reference MSE. The seven axial slices used for the healthy volunteers covered the lateral ventricles and were aligned with the subcallosal plane [97]. The target region for each MS patient was chosen individually to cover lesions, which were first identified using 3D *T*_2_‐weighted SPACE MRI.

A test–retest experiment was conducted in two subjects with extended brain coverage (27 slices). The same imaging protocol was repeated consecutively within the same session without repositioning, allowing assessment of the intrinsic measurement repeatability [98, 99] of the proposed approach.

### 2.10 Quantitative Analysis

For LOTUS shimming validation, the standard deviation (SD) of the frequencies of the resolved *B*_0_ maps obtained from the EPI module after nonlinear model‐based reconstruction was calculated for the skull‐stripped whole brain and for a target region in the occipital lobe. To evaluate the impact of improved *B*_0_ shimming on *T*_2_* mapping, the coefficient of variation (CoV = SD/mean) was calculated for 12 regions of interest (ROIs, 5 × 5 pixels).

Scatter and Bland–Altman plots were used to compare ROI‐based mean quantitative values derived from $T_{2},\;T_{2}^{*},\;\text{and}\;R_{2}^{\prime }$ maps obtained using the reference methods and the proposed approach (scatter plots: identity line is dashed, regression line is blue; Bland–Altman plots: mean difference indicated in blue, 95% limits of agreement (LoA) indicated by dashed red lines). The Pearson correlation coefficient (*R*) and coefficient of determination (*R* ^2^) are annotated on the scatter plots, while the mean difference (bias) and 95% LoA are annotated on the Bland–Altman plots. The results from Bland–Altman analyses are reported as mean ± SD of the differences.

For the phantom analysis, 10 ROIs (11 × 11 pixels) were selected. Eight ROIs were located in the agar vials, and two ROIs were placed in the main body of the phantom. For the subjects, 77 ROIs were manually selected per subject (5 × 5 pixels, 11 ROIs per slice). These ROIs were selected across various brain regions, including deep gray matter, and frontal and occipital white matter (WM). Care was taken to avoid vascular and CSF‐containing regions and areas affected by off‐resonance artifacts in the *T*_2_* maps. This small ROI size was chosen to minimize partial‐volume effects and to enable placement within small regions, for example, globus pallidus. For the repeatability analysis, 270 ROIs (5 × 5 pixels, 10 ROIs per slice) were selected using the same criteria. For the retrospective undersampling experiment, voxel‐wise absolute error maps were calculated relative to the CG‐SENSE reference. In addition, the mean absolute percentage error (MAPE) was computed across all voxels in the error maps, after applying a binary mask to exclude CSF regions. Before quantitative analyses, quantitative maps were coregistered using rigid 2D registration (details in Supporting Information S2).

## 3 Results

### 3.1 Phantom Validation

**FIGURE 2:**
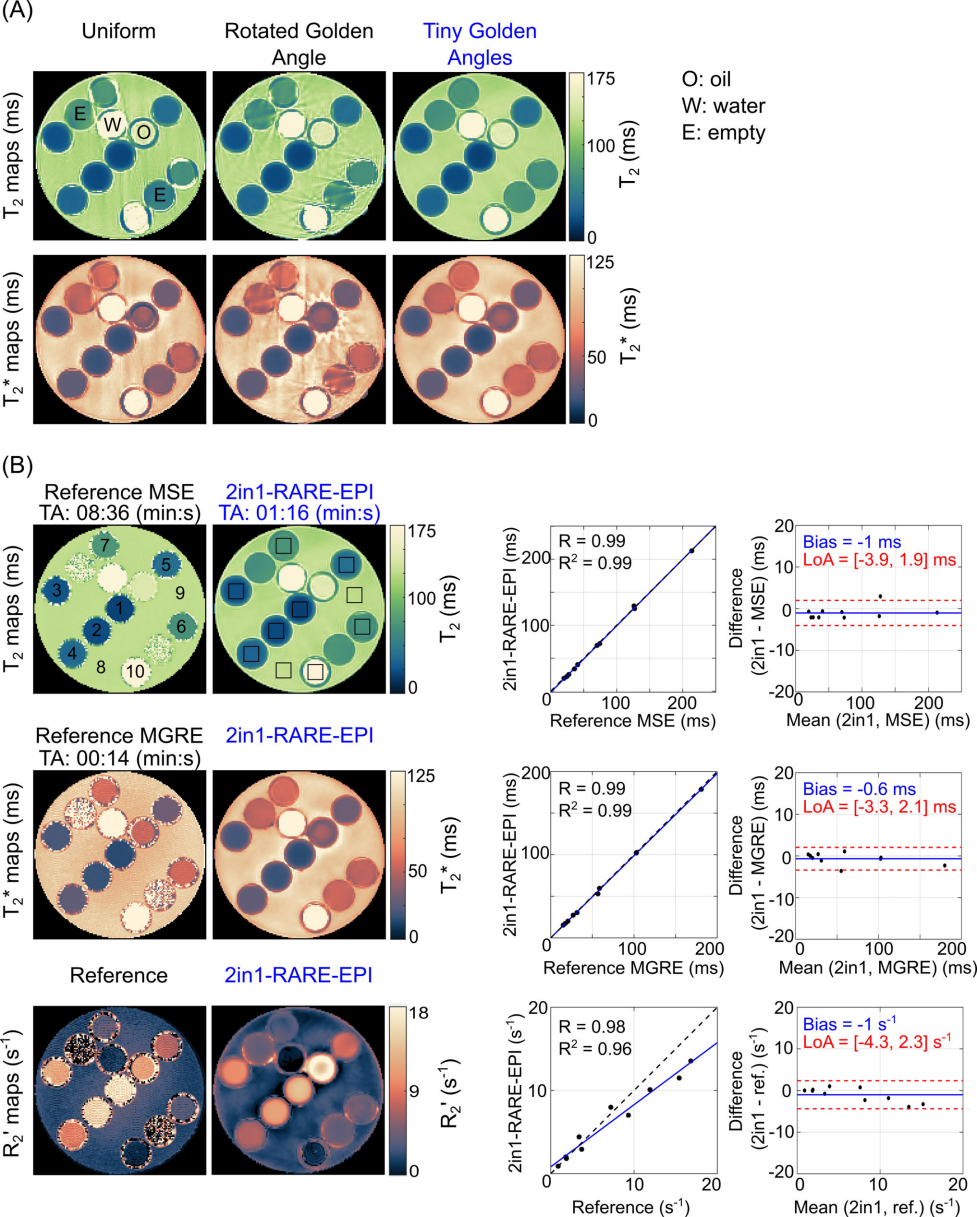
Phantom validation of *T*_2_, *T*_2_*, and *R*_2_′ mapping with 2in1‐RARE‐EPI and model‐based reconstruction. (A) *T*_2_ and *T*_2_* maps reconstructed from data acquired with three radial *k*‐space trajectories: uniform angle, rotated golden angle, and tiny golden angles. The tiny golden angles strategy minimizes streaking artifacts around the oil‐ and water‐filled vials, and the two empty vials. (B) Model‐based estimated *T*_2_, *T*_2_*, and *R*_2_′ maps derived from accelerated 2in1‐RARE‐EPI are in agreement with the fully sampled Cartesian reference methods. Scatter plots demonstrate strong linear correlations (*R* ≥ 0.98), and Bland–Altman plots showed mean differences of −1 ± 1.5 ms, −0.6 ± 1.4 ms, and −1 ± 1.7 s^−1^ for *T*_2_, *T*_2_*, and *R*_2_′, respectively. Acquisition times (TA) for 2in1‐RARE‐EPI and the reference method are indicated. The mean differences (bias) and 95% limits of agreement (LoA) are annotated in the Bland–Altman plots.

$T_{2}\;{\text{and}\;T}_{2}^{*}$ maps were obtained from accelerated 2in1‐RARE‐EPI and model‐based reconstruction of uniform, rotated golden angle, and tiny golden angle *k*‐space sampling schemes (Figure 2A). The tiny golden angle scheme minimized the impact of off‐resonance effects. Model‐based $T_{2},\;T_{2}^{*},\text{and}\;R_{2}^{\prime }$ maps derived from accelerated 2in1‐RARE‐EPI were in agreement with the fully sampled reference methods MSE and MGRE (Figure 2B). Scatter plots showed $R\geq 0.98,R^{2}\geq 0.96$ across $T_{2},\;T_{2}^{*},\;\text{and}\;R_{2}^{\prime }$ mapping. Bland–Altman analysis showed mean differences of $-1\pm 1.5\;\mathrm{ms},-0.6\pm 1.4\;\mathrm{ms},\text{and}-1\pm 1.7s^{-1}$ for $T_{2},\;T_{2}^{*},\;\text{and}\;R_{2}^{\prime }$, respectively.

### 3.2 RARE‐Informed Initialization for *T*_2_* Mapping

**FIGURE 3:**
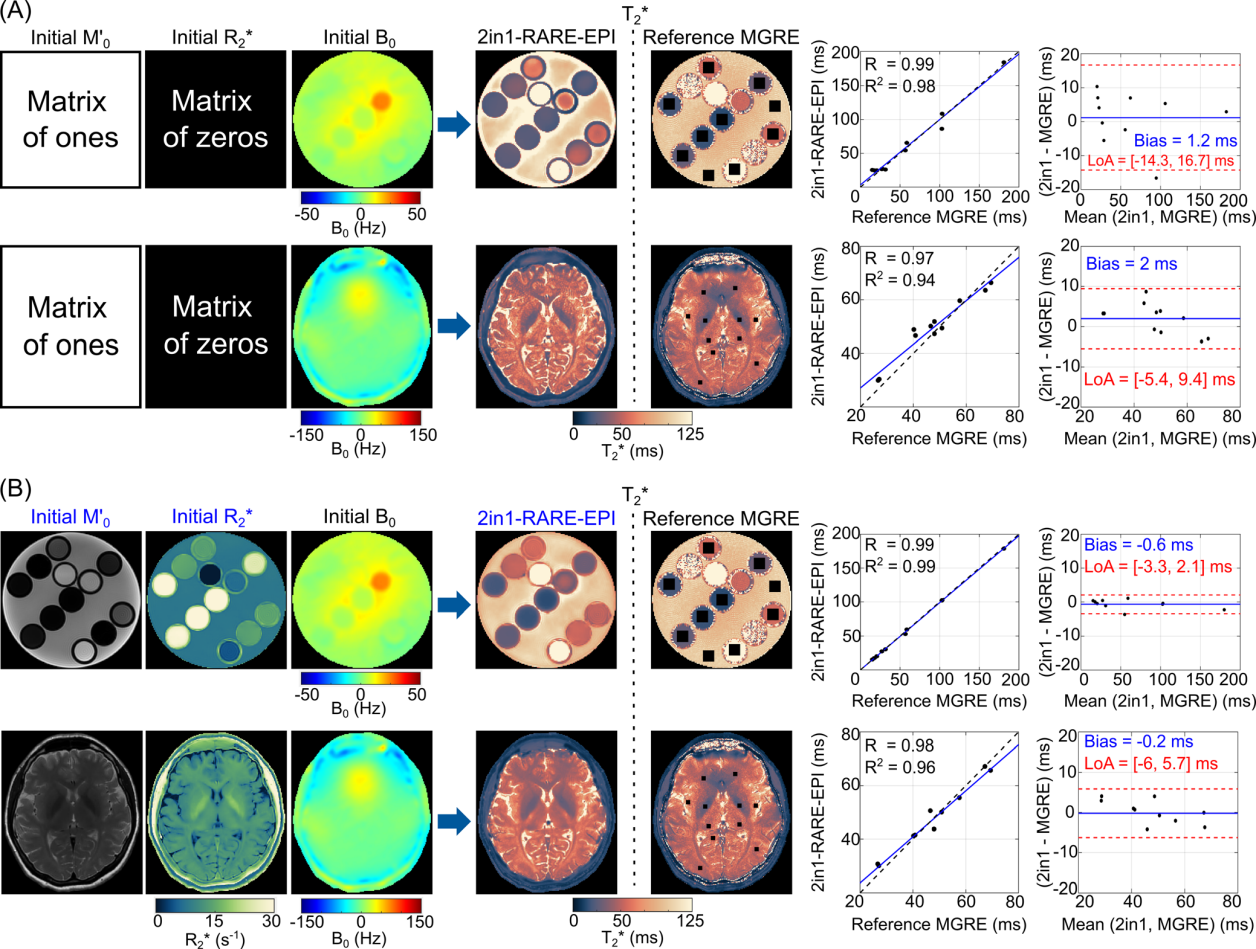
Initialization strategies for solving the nonlinear inverse problem for *T*_2_* mapping. (A) Conventional initialization. *M*′_0_ and *R*_2_* maps are initialized with ones (scaled by 0.1) and zeros, respectively. (B) Proposed initialization. The inverse problem for the RARE module is solved first. Then, *M*′_0_ is initialized with a synthetic last RARE echo image and *R*_2_* is initialized with the *R*_2_ map. An initial *B*_0_ estimate is derived from early EPI echoes in both strategies. *T*_2_* maps obtained from both initialization strategies are compared to the fully sampled reference. The proposed initialization demonstrates improved correlation and reduced bias and variability, as shown in the corresponding scatter and Bland–Altman plots.

The impact of proper initialization for solving the nonlinear inverse problem for *T*_2_* mapping is highlighted in Figure 3 using both phantom and human data. In the absence of prior information, $M_{0}^{\prime }$ and *R*_2_* are initialized with ones (scaled by 0.1) and zeros, respectively [45] (Figure 3A). In contrast, our proposed initialization exploits information from the RARE reconstruction. $M_{0}^{\prime }$ is initialized with a synthetic last RARE echo image and *R*_2_* with a scaled *R*_2_ map (Figure 3B). For both cases, an initial *B*_0_ estimate is deduced from early EPI echoes [87]. When both initialization strategies were compared to the MGRE‐*T*_2_* reference, our proposed approach yielded reduced bias and variability: mean difference improved from 1.2±7.9to−0.6±1.4ms in phantom data, and from 2±3.8to−0.2±3ms in human data. The RARE‐informed initialization also yields faster convergence of the nonlinear optimizer (Figure S5).

### 3.3 LOTUS Shimming Improved *B*_0_ Field Homogeneity

**FIGURE 4:**
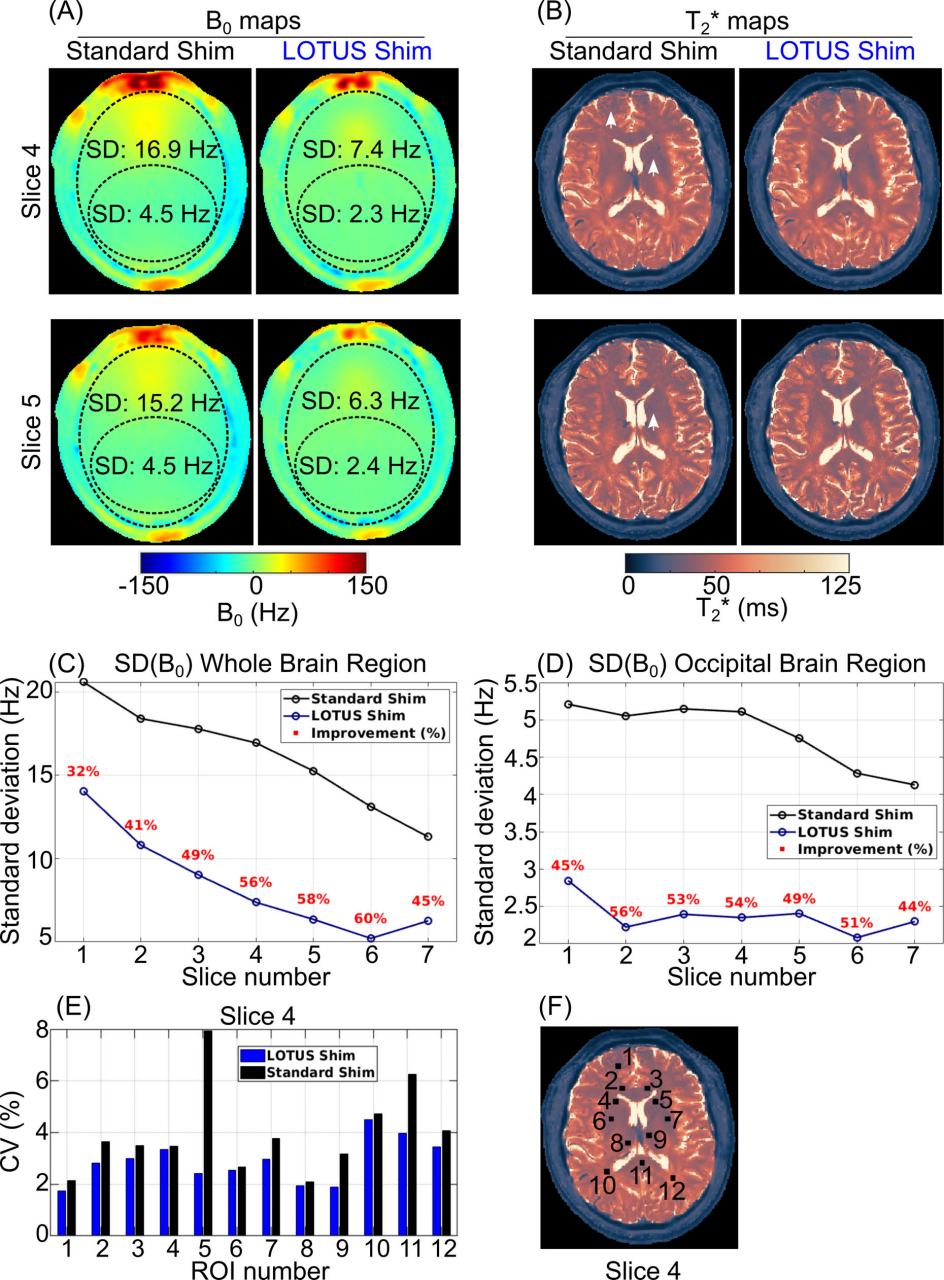
(A) *B*_0_ and (B) *T*_2_* maps of a healthy subject obtained with 2in1‐RARE‐EPI and model‐based reconstruction, using standard and LOTUS shimming. Two representative slices are shown. LOTUS yields lower standard deviation (SD) of the frequency dispersion compared to standard shimming for the whole (skull‐stripped) brain and for the occipital brain region. *T*_2_* maps obtained using standard shimming reveal regions with artificially shortened *T*_2_* (arrows), which are resolved after applying LOTUS shimming. (C) LOTUS consistently reduces the SD of *B*_0_ across seven slices compared to standard shimming, indicating improved *B*_0_ homogeneity. (D) A similar trend is observed in the occipital brain ROI. (E) The improvement in *B*_0_ homogeneity using LOTUS is reflected in the *T*_2_* maps, which show consistently lower coefficients of variation for the analyzed ROIs located in slice 4, shown in (F).

Prior to the phantom and human studies, we examined the enhancement of *B*_0_ uniformity facilitated by LOTUS shimming. Figure 4A,B shows *B*_0_ and *T*_2_* maps obtained for two slices of a healthy volunteer using 2in1‐RARE‐EPI with model‐based reconstruction. LOTUS shimming facilitated reduced frequency dispersion for ROIs encompassing the whole brain and the occipital brain region, indicating improved *B*_0_ field homogeneity. *T*_2_* maps obtained after standard shimming revealed regions with artificially shortened *T*_2_* values (Figure 4B, white arrows). These artifacts were resolved with LOTUS. LOTUS improved *B*_0_ homogeneity by 32%–60% compared to standard shimming, based on the SD of *B*_0_ across the entire brain slice (Figure 4C). For an ROI encompassing the occipital lobe, LOTUS yielded a 44%–56% improvement in *B*_0_ homogeneity relative to standard shimming (Figure 4D). *T*_2_* mapping supported by LOTUS shimming provided consistently lower CoV (Figure 4E) for 12 ROIs in the white and gray matter (Figure 4F).

### 3.4 Human Study: Healthy Subjects

**FIGURE 5:**
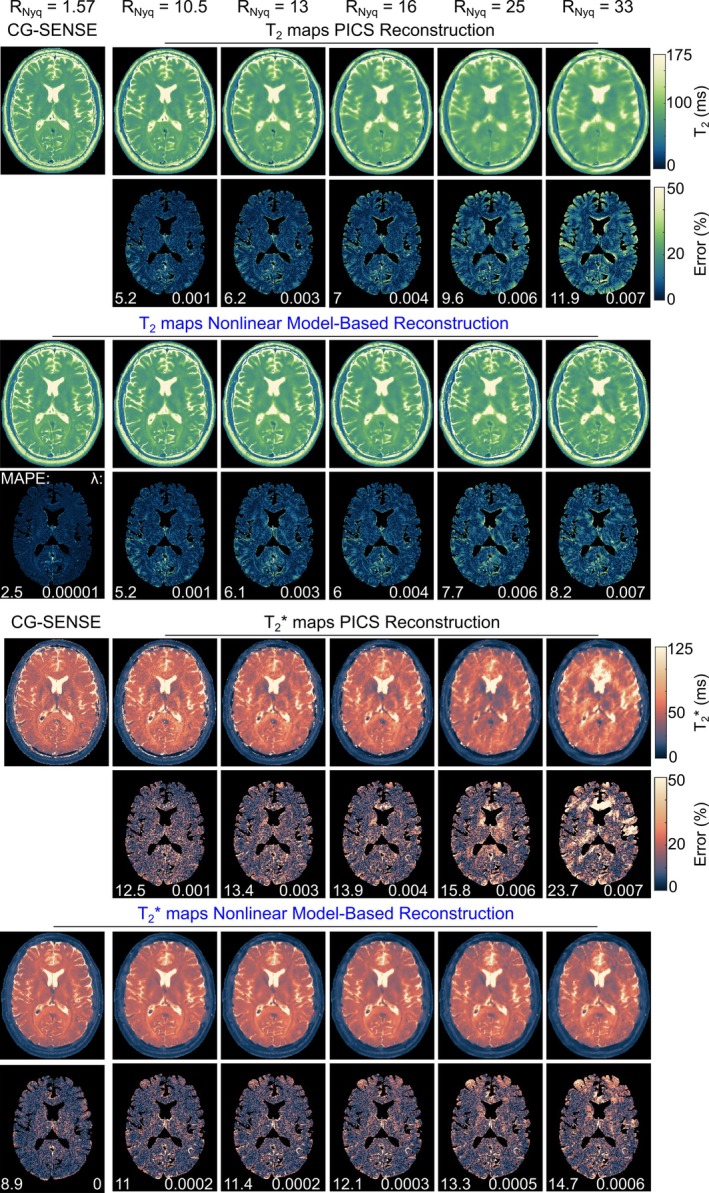
Comparison of nonlinear model‐based reconstruction versus PICS reconstruction for *T*_2_ and *T*_2_* mapping of accelerated 2in1‐RARE‐EPI data. Five different acceleration factors ($R_{\mathrm{Nyq}}$) were investigated. Quantitative maps obtained from 256 RF excitations ($R_{\mathrm{Nyq}}=1.57$) with CG‐SENSE reconstruction were used as the reference and are shown in column 1. Rows 1 and 5: *T*_2_ and *T*_2_* maps obtained from PICS reconstruction. Rows 2 and 6: corresponding absolute difference maps relative to the CG‐SENSE reference for PICS reconstruction. Rows 3 and 7: *T*_2_ and *T*_2_* maps derived from the nonlinear model‐based reconstruction. Rows 4 and 8: corresponding absolute difference maps relative to the CG‐SENSE reference for model‐based reconstruction. The mean absolute percentage error (MAPE, %) and the regularization parameter value (*λ*) used in each reconstruction are annotated on the error maps. For nonlinear model‐based reconstructions, *λ* refers to the minimum regularization value reached during the Newton iterations. A binary mask excluding the CSF was used to focus the error analysis on the brain parenchyma.

Figure 5 illustrates the comparison of $T_{2}\;\text{and}\;T_{2}^{*}$ maps between model‐based and PICS reconstructions under retrospective undersampling. The CG‐SENSE *T*_2_ map exhibits streaking artifacts arising from the straight sinus, and the CG‐SENSE *T*_2_* map exhibits streaking artifacts in the frontal lobe and ringing artifacts along the cortical boundary. These artifacts are minimized in both model‐based quantitative maps up to $R_{\mathrm{Nyq}}=16$, with only minor reduction of high spatial frequency information. Error maps (up to $R_{\mathrm{Nyq}}=16$) show low, spatially unstructured errors across the brain, with increased errors near tissue‐CSF boundaries, reflecting differences in partial‐volume handling by both approaches.

Model‐based *T*_2_ maps showed accuracy comparable to PICS when measured against the CG‐SENSE reference while better preserving high spatial frequency information across all acceleration factors.

Model‐based *T*_2_* maps showed reduced blurring, less noise, and fewer streaking artifacts than the PICS‐based *T*_2_* maps across all acceleration factors. The MAPE, measured relative to the CG‐SENSE reference, was consistently lower for model‐based *T*_2_ and *T*_2_* maps across all acceleration factors.

**FIGURE 6:**
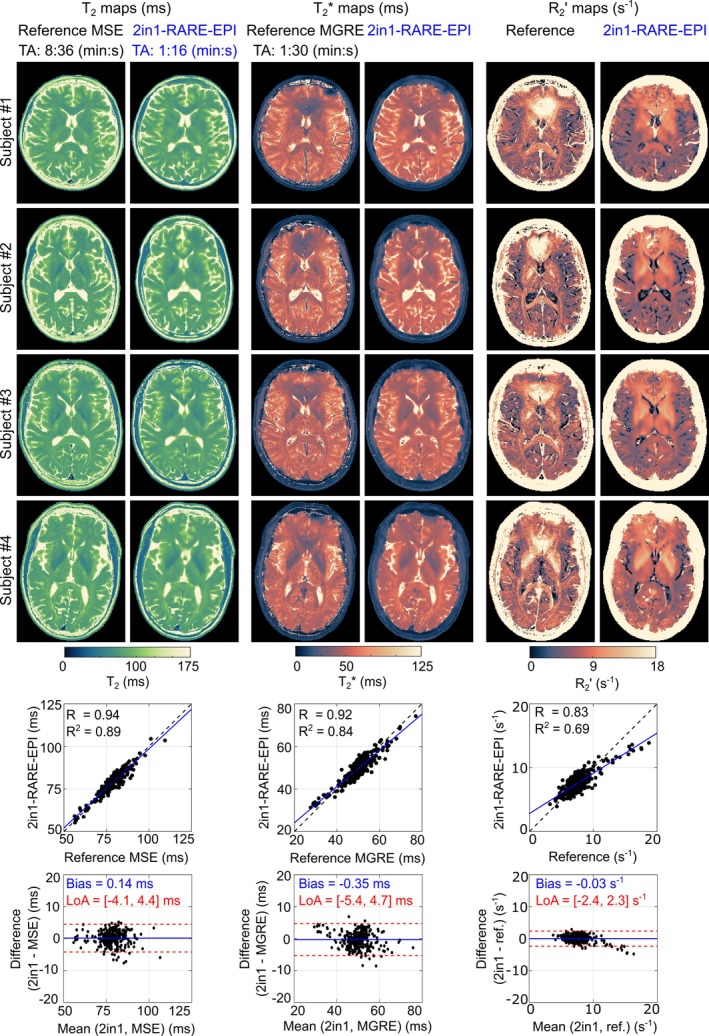
In vivo validation (part 1). *T*_2_, *T*_2_*, and *R*_2_′ maps from four healthy volunteers (first two rows: females; last two rows: males) obtained using model‐based reconstruction of accelerated 2in1‐RARE‐EPI are compared with fully sampled MSE and MGRE references. One representative slice is shown for each subject. With the proposed approach, the acquisition time (TA) is reduced from 10:06 to 1:16 min:s, representing an eightfold scan‐time acceleration. The reference *T*_2_* and *R*_2_′ maps exhibit artifacts in the frontal horns of the lateral ventricles due to off‐resonance effects, which are mitigated with our approach. The resulting parametric maps are in agreement with the fully sampled MSE and MGRE references. Scatter plots demonstrate strong linear correlations (*R* ≥ 0.83), and Bland–Altman plots showed mean differences of 0.14 ± 2.2 ms, −0.35 ± 2.6 ms, and −0.03 ± 1.2 s^−1^ for *T*_2_, *T*_2_*, and *R*_2_′, respectively. The plots include 308 ROIs (4 subjects × 7 slices per subject × 11 ROIs per slice).

**FIGURE 7:**
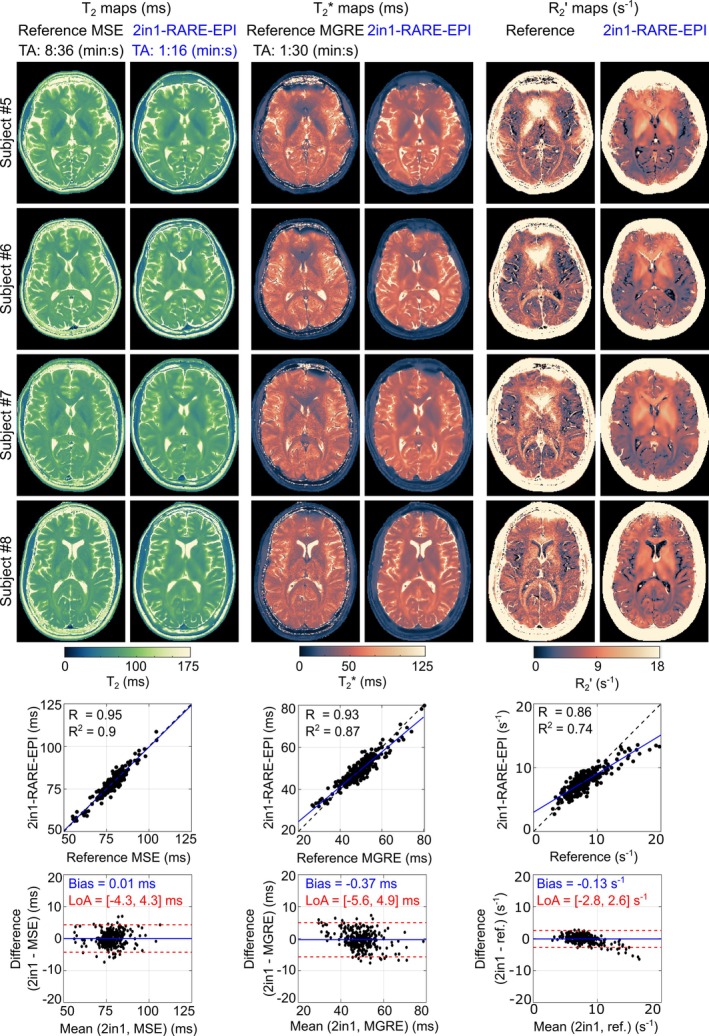
In vivo validation (part 2). *T*_2_, *T*_2_*, and *R*_2_′ maps from four additional healthy volunteers (first two rows: females; last two rows: males) obtained using model‐based reconstruction of accelerated 2in1‐RARE‐EPI are compared with fully sampled MSE and MGRE references. The findings are consistent with those detailed in Figure 6. Scatter plots demonstrate strong linear correlations (*R* ≥ 0.86), and Bland–Altman plots showed mean differences of 0.01 ± 2.2 ms, −0.37 ± 2.7 ms, and −0.13 ± 1.4 s^−1^ for *T*_2_, *T*_2_*, and *R*_2_′, respectively. The plots include 308 ROIs (4 subjects × 7 slices per subject × 11 ROIs per slice).

We investigated eight healthy volunteers, comparing $T_{2},\;T_{2}^{*},\;\text{and}\;R_{2}^{\prime }$ maps from accelerated 2in1‐RARE‐EPI ($R_{\mathrm{Nyq}}=10.5$) against the fully sampled MSE and MGRE references (Figures 6 and 7). Reference *T*_2_* and *R*_2_′ maps exhibited off‐resonance‐related artifacts in the frontal horns of the lateral ventricles that were minimized in 2in1‐RARE‐EPI maps. The parametric maps obtained with our approach were consistent with the references, while reducing the acquisition time from 10:06 to 1:16 min:s, corresponding to an 8‐fold scan‐time acceleration. Quantitative agreement was confirmed by the scatter and Bland–Altman plots embedded in Figures 6 and 7. In each figure, the plots include 308 ROIs (4 subjects × 7 slices per subject × 11 ROIs per slice). Scatter plots showed *R* ≥ 0.94, *R* ^2^ ≥ 0.89 for *T*_2_ mapping, and *R* ≥ 0.92, *R* ^2^ ≥ 0.84 for *T*_2_* mapping. For *R*_2_′ mapping, correlations were lower (*R* ≥ 0.83, *R* ^2^ ≥ 0.69), likely due to *R*_2_′ overestimation in the globi pallidi by the MGRE reference. The *R*_2_′ Bland–Altman outliers arise from these ROIs, where residual *B*_0_ gradients in the MGRE reference lead to enhanced spin dephasing. The Bland–Altman plots showed absolute mean differences below $0.14\;\mathrm{ms},0.37\;\mathrm{ms},\text{and}\;0.13\;s^{-1}$ for $T_{2},\;T_{2}^{*},\text{and}\;R_{2}^{\prime }$, respectively. Individual subject plots are provided in Figure S6. Videos S1–S8 show $T_{2},\;T_{2}^{*},\;\text{and}\;R_{2}^{\prime }$ maps, together with absolute error maps relative to the references and the corresponding ROI locations, for subjects #1–8. Model‐based *B*_0_ maps are also shown in the videos.

**FIGURE 8:**
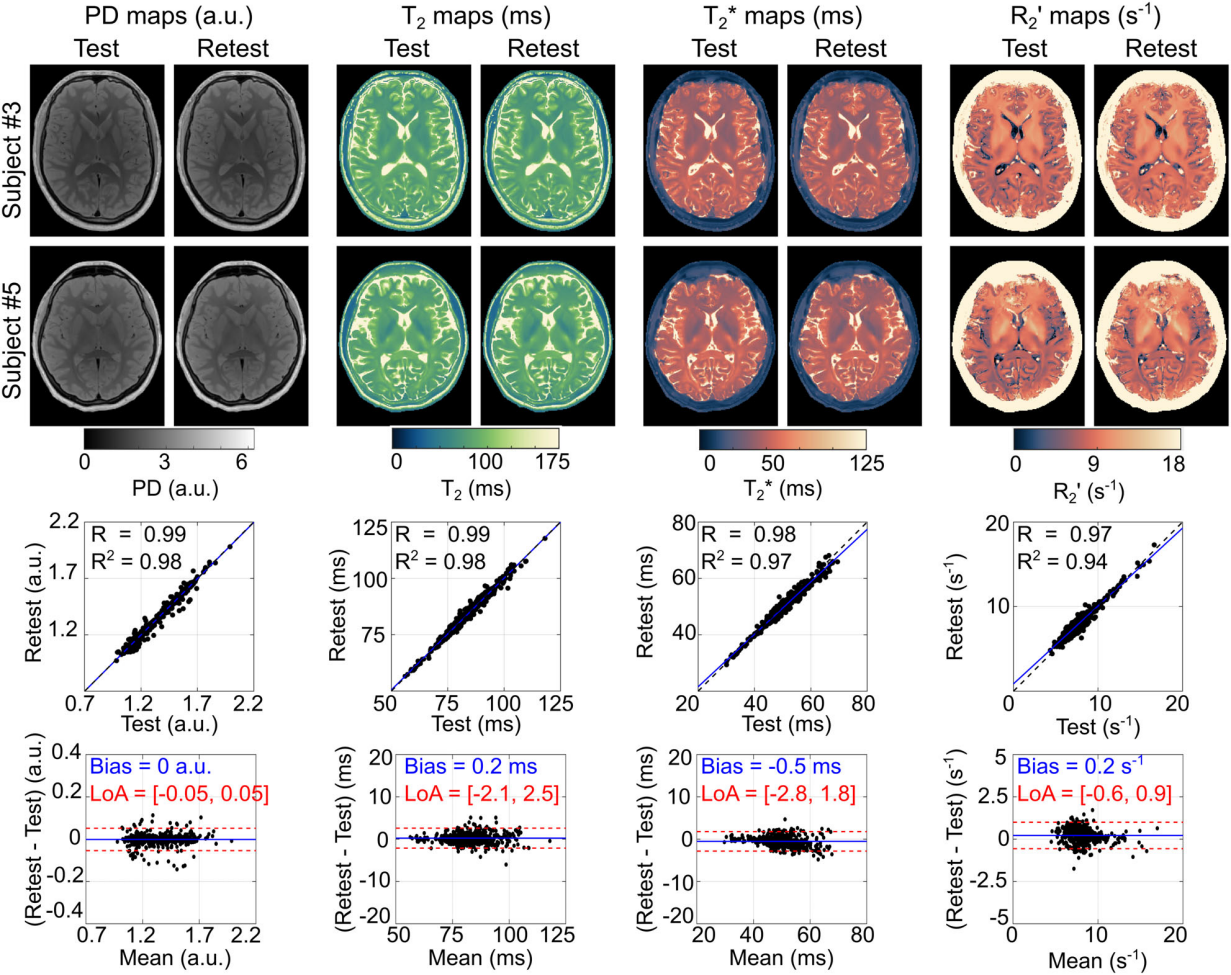
Repeatability assessment of 2in‐RARE‐EPI with model‐based reconstruction. PD, *T*_2_, *T*_2_*, and *R*_2_′ maps for one representative slice (out of 27) are shown for two subjects. Statistical analysis of 540 ROIs per subject (5 × 5 pixels, 2 subjects × 27 slices per subject × 10 ROIs per slice) shows a strong correlation (scatter plots) and a small bias (Bland–Altman plots) between test and retest scans for both subjects and for all four quantitative maps.

Figure 8 shows repeated $\mathrm{PD},\;T_{2},\;T_{2}^{*},\text{and}\;R_{2}^{\prime }$ maps from one representative slice (out of 27) in two subjects. Analysis of 540 ROIs (270 per subject) demonstrated test–retest agreement across all quantitative maps (*R* ≥ 0.97, *R* ^2^ ≥ 0.94). Across the two subjects, the mean differences were: 0±0.02a.u. for PD, 0.2±1.2ms for *T*_2_, −0.5±1.1ms for *T*_2_*, and $0.2\pm 0.39\;s^{-1}$ for *R*_2_′. Videos S9 (subject #3) and S10 (subject #5) show $\mathrm{PD},\;T_{2},\;T_{2}^{*},\;\text{and}\;R_{2}^{\prime }$ maps, as well as absolute error maps relative to the test scan, and the corresponding ROI locations, across all 27 slices. The acquisition time of 2in1‐RARE‐EPI for 27 slices was 3:15 min:s (TR = 5150 ms), whereas the reference methods would require 24:16 min:s for the same slice coverage (18:29 min:s for MSE and 5:47 min:s for MGRE), corresponding to a 7.5‐fold scan‐time acceleration.

### 3.5 Human Study: MS Patients

**FIGURE 9:**
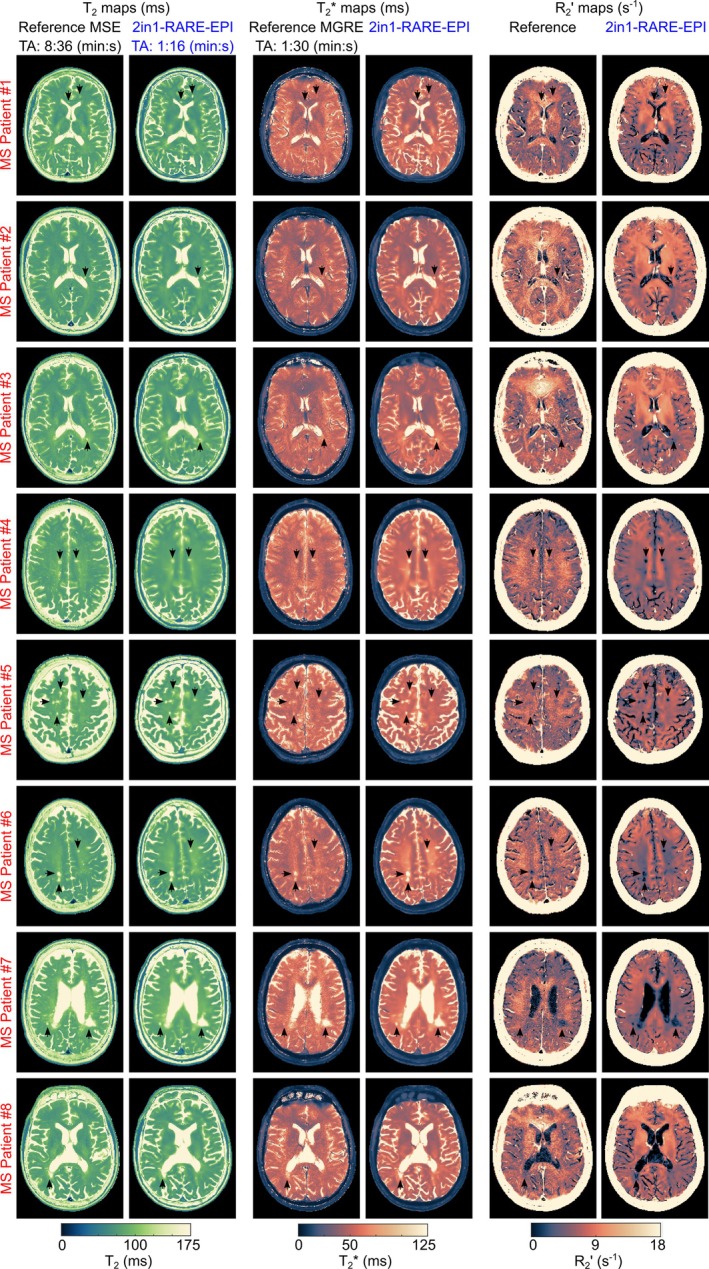
Feasibility study in patients with multiple sclerosis (MS). Small lesions (arrows) in patients #1–3 are clearly depicted in the *T*_2_, *T*_2_*, and *R*_2_′ maps derived from accelerated 2in1‐RARE‐EPI and in the *T*_2_ map from the MSE reference. In contrast, conventional *T*_2_* and *R*_2_′ mapping show limited sensitivity for detecting these lesions. Mid‐sized lesions (patients #4–6) and large confluent lesions (patients #7 and #8) are well delineated in the *T*_2_, *T*_2_*, and *R*_2_′ maps derived from both accelerated 2in1‐RARE‐EPI and the reference methods (MSE and MGRE).

**FIGURE 10:**
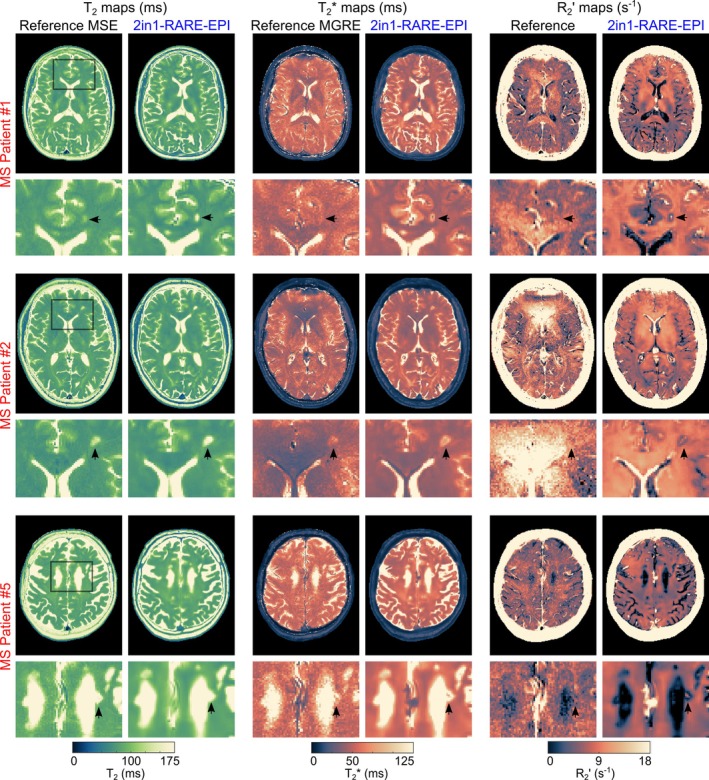
Detection of the central vein sign with 2in1‐RARE‐EPI in MS patients. *T*_2_ maps from both the accelerated 2in1‐RARE‐EPI with model‐based reconstruction and the fully sampled Cartesian MSE reference clearly depict lesions (arrows) in three representative MS patients, reflecting comparable sensitivity to focal lesion detection. In contrast, the proposed method further enables clear visualization of intralesional veins on the corresponding *T*_2_* and *R*_2_′ maps, appearing as a centrally located high‐susceptibility structure in the lesion of patient #1 and as a vein running centrally through the long axis of the lesion in patients #2 and #5. This feature is difficult to visualize in the fully sampled reference *T*_2_* and *R*_2_′ maps.

The feasibility of accelerated 2in1‐RARE‐EPI for detecting focal brain lesions was demonstrated in eight MS patients (Figure 9). Small lesions in patients #1–3 are well depicted across all three 2in1‐RARE‐EPI maps ($T_{2},\;T_{2}^{*},\;R_{2}^{\prime }$) and in the MSE‐*T*_2_ reference, but are difficult or impossible to detect with conventional $T_{2}^{*}\;\text{and}\;R_{2}^{\prime }$ mapping. Patient #1 demonstrates lesions in the corpus callosum and deep WM. Patient #2 shows a periventricular lesion in the posterior horn of the left lateral ventricle; patient #3 exhibits a WM lesion near the same posterior region. Mid‐size lesions in patients #4–6 are well delineated in the $T_{2},\;T_{2}^{*},\text{and}\;R_{2}^{\prime }$ maps from both 2in1‐RARE‐EPI and the reference methods. Patient #4 has bilateral periventricular lesions located along the superior aspect of the lateral ventricles, while patients #5–6 exhibit distinct WM lesions. Large confluent lesions in patients #7–8 are likewise well delineated in both approaches. Additionally, the WM lesion of patient #1 shows a centrally located high‐susceptibility structure, consistent with the MS‐specific central vein sign [96, 100, 101], in the 2in1‐RARE‐EPI $T_{2}^{*}\;\text{and}\;R_{2}^{\prime }$ maps. Figure 10 shows a magnified view of this feature, together with two additional examples from patients #2 and #5, in which a vein running along the long axis of the lesions is visualized.

## 4 Discussion

We demonstrated that 2in1‐RARE‐EPI with nonlinear model‐based reconstruction provides $T_{2},\;T_{2}^{*},\;\text{and}\;R_{2}^{\prime }$ maps consistent with the reference methods, while achieving a 7.5‐fold scan‐time acceleration. It produced fewer artifacts than CG‐SENSE, and maintained spatial detail and accuracy more consistently than PICS at higher acceleration factors. The proposed framework eliminates the need for additional calibration scans because gradient delay errors are corrected in an auto‐calibrated preprocessing step, and coil sensitivity and *B*_0_ maps are estimated during reconstruction.

Our approach enabled clearer visualization of lesions and their internal structure in the *T*_2_* and *R*_2_′ maps compared to the MGRE reference, enabling detection of the clinically relevant central vein sign [102] and thus adding diagnostic specificity by distinguishing MS lesions from mimics [96]. This improvement is likely caused by the different weighting of the two acquisitions. Because 2in1‐RARE‐EPI estimates *T*_2_* under high *T*_2_‐weighting (from the RARE module) and low T_1_‐weighting (long TR = 2 s), the available signal is higher in tissues with long $T_{1}\;\text{and}\;T_{2}$, such as WM lesions, improving the stability and accuracy of *T*_2_* estimation in these regions. In contrast, the MGRE reference is strongly T_1_‐weighted (short TR = 50 ms) and the signal is enhanced in short‐T_1_ tissues and suppressed in long‐T_1_ tissues such as WM lesions. This difference in weighting may particularly affect regions with partial‐volume effects and highly ordered structures with multiexponential decay, such as the splenium. In these regions, the MGRE *T*_2_* may be biased downward by the short‐T_1_, short‐*T*_2_ myelin water compartment, whereas 2in1‐RARE‐EPI *T*_2_* may be more influenced by intra‐ and extracellular water with longer T_1_ and *T*_2_, which may contribute to higher *T*_2_* and correspondingly lower *R*_2_′. A longer TR in the MGRE acquisition would reduce T_1_‐weighting and increase the available signal in long‐T_1_ tissues, which may improve lesion depiction and the stability of *T*_2_* estimation in such regions. Other modern acquisition strategies for simultaneous $T_{2}\;\text{and}\;T_{2}^{*}$ mapping like MR fingerprinting [25], also estimate *T*_2_* at high T_1_‐weighting, potentially reducing *T*_2_* contrast for long‐T_1_ features like WM lesions.

The improved visualization of MS lesions and the central vein sign with our approach also reflects the synergy between the acquisition and the nonlinear model‐based reconstruction. In particular, the proposed *k*‐space trajectory can make off‐resonance‐related artifacts more localized and less structured, as seen in the phantom experiment, while the nonlinear model‐based reconstruction explicitly accounts for *B*_0_‐related phase evolution and further reduces off‐resonance artifacts that might otherwise obscure these clinically relevant features. For instance, as shown in Figure S9, PICS reconstruction of 2in1‐RARE‐EPI data exhibits reduced visibility of MS lesions and the central vein sign in the *T*_2_* and *R*_2_′ maps compared to nonlinear model‐based reconstruction, particularly in regions affected by off‐resonance effects.

Related approaches require calibration scans. EPTI20 requires *B*_0_ and coil sensitivity calibration, BUDA‐SAGE [18] needs a coil sensitivity prescan, and MR fingerprinting needs a prescan for gradient delay correction. In contrast, with our approach, radial acquisition combined with auto‐calibrated gradient delay correction and calibrationless model‐based reconstruction eliminates the need for separate calibration scans, thereby avoiding potential errors arising from prescan miscalibrations. Although the proposed model‐based reconstruction increases numerical complexity, it enables optimal use of all acquired data through joint parameter estimation. For example, while phase‐singularity‐related artifacts are relatively rare, the model‐based approach mitigates such artifacts by exploiting information from all multiecho data (Figure S7). In contrast, other approaches typically estimate coil sensitivities from the first echo only, as the simple combination of multiecho data for coil estimation is suboptimal due to echo‐dependent phase evolution and contrast differences. As phase singularities may still arise in nonlinear model‐based reconstructions, future work will investigate the integration of strategies for the detection and correction of phase singularities in coil sensitivity maps [103].

Compared with existing Cartesian methods, the proposed radially sampled technique is inherently more robust to motion. Motion‐induced errors are distributed incoherently in *k*‐space, producing streaking artifacts, which can be mitigated via sparsity regularization [50, 69]. Continuous oversampling of the *k*‐space center provides a signal‐averaging effect [69], further reducing motion artifacts, while also enabling self‐navigation [104] through the repeatedly sampled center of *k*‐space. Despite these features, severe motion may still lead to artifacts, and future work will focus on integrating explicit motion compensation mechanisms into our framework. For instance, this self‐navigated radial sampling offers the opportunity to formulate a consistency criterion [105] to discard or compensate motion‐corrupted spokes.

Regarding acquisition time, the proposed 2in1‐RARE‐EPI sequence requires 3:15 min:s to acquire 27 slices at a spatial resolution of 1 × 1 × 3 mm^3^ at $R_{\mathrm{Nyq}}=10.5$. This is longer than the multislice EPTI [19] approach, which requires approximately 1 min for 34 slices at the same resolution. The longer acquisition time of the proposed method is partially influenced by specific absorption rate (SAR) constraints. In addition, the acceleration factor of $R_{\mathrm{Nyq}}=10.5$ for the human validation was chosen conservatively to prioritize robust quantitative mapping, reliable lesion depiction, and sufficient signal‐to‐noise ratio (SNR) across subjects and patients. Further acceleration strategies could be employed to reduce the acquisition time. These include, but are not limited to, optimizing the flip‐angle train in the spin‐echo sequence to reduce SAR, incorporating simultaneous multislice techniques, and leveraging deep‐learning‐enhanced model‐based reconstruction [106].

Nonlinear model‐based reconstruction is computationally more demanding than conventional reconstruction methods, a limitation shared by other advanced approaches such as 3D‐EPTI [20], which employs subspace reconstruction followed by dictionary matching. 3D‐EPTI requires approximately 3 h on a single GPU to reconstruct a 210 mm slab volume. Using the proposed approach and assuming the same volume, approximately 70 slices of 3 mm would be required, resulting in an estimated total reconstruction time of about 2.6 h (˜132 s per slice on a single GPU), which is on the same order as 3D‐EPTI. This reported runtime reflects a sequential implementation with conservative parameter settings to ensure robust convergence. In practice, the proposed approach enables straightforward parallelization across slices, while the model‐based framework allows efficient multiGPU execution within each slice. Reducing the amount of data handled by the reconstruction algorithm can further reduce reconstruction time [107]. For example, by reducing the grid oversampling ratio from 2 to 1.5 and the number of virtual coils from 12 to 10, reconstruction time per slice is expected to decrease from 132 to 79 s. Further acceleration is feasible through improved preconditioning to reduce iteration counts [108, 109], as well as through improved initialization strategies that accelerate convergence [110]. Despite the longer reconstruction time for quantitative maps, the proposed 2in1‐RARE‐EPI sequence can provide immediate feedback to the operator. High‐resolution *T*_2_‐ and *T*_2_*‐weighted images can be reconstructed within 4 s by combining a subset of echoes, allowing rapid verification of scan quality before generating quantitative maps (see Figure S8).

The repeatability of *R*_2_′ was lower than $T_{2}\;\text{and}\;T_{2}^{*}$ (Videos S9 and S10). This is expected because *R*_2_′ is derived indirectly from $T_{2}\;\text{and}\;T_{2}^{*}$, such that small variations in these parameters can propagate and lead to amplified errors in *R*_2_′. In addition, a spatial variability in the relative error maps for *R*_2_′ is observed, with higher relative differences particularly in cortical and posterior regions. Because baseline *R*_2_′ values are lower in such regions, even modest absolute differences can correspond to large percentage errors. Residual spatially varying effects, including ringing artifacts and minor coregistration inaccuracies between test and retest scans, may further contribute to this variability, with their impact amplified in *R*_2_′. In iron‐rich subcortical regions, *R*_2_′ values are higher, and modest absolute differences correspond to small percentage errors.

Another limitation of the current 2in1‐RARE‐EPI implementation is that it does not provide reliable $T_{2}\;\text{and}\;T_{2}^{*}$ estimates in CSF because the set of echo times was optimized for brain parenchyma [30]. This limitation is shared by most techniques designed for simultaneous $T_{2}\;\text{and}\;T_{2}^{*}$ mapping. In addition, the long TR required to minimize T_1_ effects makes our approach less suitable for dynamic or time‐resolved applications, for which single‐shot strategies are more appropriate [23, 24]. Currently, stimulated echoes arising from $B_{1}^{+}$ inhomogeneity are not explicitly modeled, which can lead to *T*_2_ overestimation. Although this introduces bias, the resulting *T*_2_ maps remain informative for assessing relative *T*_2_ variations in many applications. Future work will incorporate RARE signal models that account for stimulated echoes while maintaining compatibility with the proposed model‐based reconstruction framework [111, 112].

Future work will include a larger and more diverse MS cohort to fully assess the clinical value of *T*_2_ and *T*_2_* mapping beyond lesion detection, including NAWM changes [1, 6, 113] and ventricular enlargement [114], as well as differentiation of MS from related demyelinating disorders such as neuromyelitis optica spectrum disorder (NMOSD) [115] and myelin oligodendrocyte glycoprotein antibody‐associated disease (MOGAD) [116]. 3D acquisition strategies for *T*_2_ mapping [117] could be extended to incorporate 2in1‐RARE‐EPI readouts, enabling whole‐brain $T_{2}\;\text{and}\;T_{2}^{*}$ mapping with improved SNR and spatial resolution, and allowing QSM reconstruction and separation of paramagnetic and diamagnetic susceptibility sources [18, 91]. Such 3D extensions of the proposed framework may benefit from 3D trajectory designs that improve quantitative parameter mapping [118], as well as from further SAR reduction achieved through optimized RF pulses [119]. Accelerated 2in1‐RARE‐EPI could be applied to other organs including the kidneys [120], where $T_{2}\;\text{and}\;T_{2}^{*}$ mapping are valuable for assessing renal oxygenation [121, 122, 123] and kidney size [124, 125, 126].

## 5 Conclusion

This study demonstrates the feasibility of combining 2in1‐RARE‐EPI with nonlinear model‐based reconstruction for accelerated, simultaneous $T_{2},\;T_{2}^{*},\;\text{and}\;R_{2}^{\prime }$ mapping, addressing key limitations of reference techniques, including long acquisition times, spatial misregistration, sensitivity to motion and off‐resonance effects, and the need for calibration scans. Clinically, the method was validated in MS patients, although the potential range of applications extends to other brain pathologies and other organs, including the heart, eyes, and kidneys. Scan time reduction facilitated by model‐based reconstruction of undersampled 2in1‐RARE‐EPI provides a technical foundation for enhanced patient comfort and compliance, and a precursor for broader clinical studies on the potential of $T_{2},\;T_{2}^{*},\;\text{and}\;R_{2}^{\prime }$ as imaging biomarkers.

## Funding

This project is funded in part by the Deutsche Forschungsgemeinschaft to T.N. (DFG, German Research Foundation, DFG‐Project number: 517901233), by the MDC‐Weizmann Helmholtz International Research School for Imaging and Data Science from the NAno to the MEso (iNAMES) to I.F.T.C. and T.N., by the National Institutes of Health (NIH) to X.W. (award numbers R01EB037186 and R01HD119407), and by the German‐Israeli Foundation for Scientific Research and Development (GIF) to S.W. (Grant No. G‐1563‐202.2/2023). The founders had no role in study design, data collection and analysis, decision to publish, or preparing the manuscript.

## Conflicts of Interest

Hoby P. Hetherington and Franz Schmitt are employees of Resonance Research Inc., Billerica, Massachusetts, USA. The other authors declare no conflicts of interest.

## Supporting information


**Figure S1:** Model‐based PD maps and magnitudes, of the first‐element coil sensitivity maps as a function of the Sobolev regularization parameters (s,l) for subject #2.
**Figure S2:** Model‐based *T*_2_ and *T*_2_* maps as a function of the regularization parameter $\lambda_{\min}$ for subject #2.
**Figure S3:** Model‐based *T*_2_* and *B*_0_ maps as a function of the Sobolev regularization parameters (s,l) for subject #2.
**Figure S4:** Top row: results from voxel‐wise fitting of the reference Cartesian MGRE data.
**Figure S5:** Normalized cost function as a function of Gauss–Newton steps for different initialization strategies for *T*_2_* mapping with nonlinear model‐based reconstruction of accelerated 2in1‐RARE‐EPI.
**Figure S6:** Scatter and Bland–Altman plots for eight healthy subjects.
**Figure S7:** Comparison of coil sensitivity maps obtained with nonlinear model‐based reconstruction and the ESPIRiT [5] method.
**Figure S8:** Weighted images reconstructed for fast feedback from the accelerated 2in1‐RARE‐EPI acquisition (left column), compared with nonlinear model‐based synthetic images (middle column) and fully sampled Cartesian reference images (right column).
**Figure S9:** Comparison of PICS and nonlinear model‐based reconstruction of 2in1‐RARE‐EPI data in MS patients.

**Videos S1–S8:** mrm70465‐sup‐0002‐Video_S1‐S8.zip. $T_{2},\;T_{2}^{*},\;\text{and}\;R_{2}^{\prime }$ maps obtained with 2in1‐RARE‐EPI and nonlinear model‐based reconstruction, and with the reference MSE and MGRE, together with absolute error maps relative to the references and the corresponding ROI locations, for subjects 1–8.

**Videos S9 and S10:** $\mathrm{PD},\;T_{2},\;T_{2}^{*},\;\text{and}\;R_{2}^{\prime }$ maps obtained with 2in1‐RARE‐EPI and nonlinear model‐based reconstruction in a test–retest experiment, for two subjects, as well as absolute error maps relative to the test scan, and the corresponding ROI locations, across all 27 slices.

## Data Availability

The human data that support the findings of this study are available on request from the corresponding author. The data are not publicly available due to privacy or ethical restrictions. Scripts for reading and preprocessing the raw data, performing nonlinear model‐based reconstruction, as well as the phantom validation data, are available at https://github.com/velasqvides/2in1‐RARE‐EPI_with_MOBAreco.
