# Supplementary material for: Simultaneous T 2, T 2*, and R 2′ Mapping for Multiple Sclerosis Using Nonlinear Model‐Based Reconstruction of Undersampled Radial RARE‐EPI MRI
Source: Magn Reson Med. 2026 Jun 18;96(4):1872–92. doi: 10.1002/mrm.70465 (PMC13419268; doi:10.1002/mrm.70465)
Supplement: Supplementary file 1 — Figure S1: Model‐based PD maps and magnitudes, of the first‐element coil sensitivity maps as a function of the Sobolev regularization parameters (s,l) for subject #2. Figure S2: Model‐based T 2 and T 2* maps as a function of the regularization parameter λmin for subject #2. Figure S3: Model‐based T 2* and B 0 maps as a function of the Sobolev regularization parameters (s,l) for subject #2. Figure S4: Top row: results from voxel‐wise fitting of the reference Cartesian MGRE data. Figure S5: Normalized cost function as a function of Gauss–Newton steps for different initialization strategies for T 2* mapping with nonlinear model‐based reconstruction of accelerated 2in1‐RARE‐EPI. Figure S6: Scatter and Bland–Altman plots for eight healthy subjects. Figure S7: Comparison of coil sensitivity maps obtained with nonlinear model‐based reconstruction and the ESPIRiT [5] method. Figure S8: Weighted images reconstructed for fast feedback from the accelerated 2in1‐RARE‐EPI acquisition (left column), compared with nonlinear model‐based synthetic images (middle column) and fully sampled Cartesian reference images (right column). Figure S9: Comparison of PICS and nonlinear model‐based reconstruction of 2in1‐RARE‐EPI data in MS patients. [file MRM-96-1872-s002.docx]

**Supporting Information**

**Simultaneous T_2_, T_2_*, and R_2_′ Mapping for Multiple Sclerosis Using Model-Based Reconstruction**

**of Undersampled Radial RARE-EPI MRI**

Jose Raul Velasquez Vides, Carl J. J. Herrmann, Thomas Gladytz, Hoby P. Hetherington, Hendrik Mattern, Xiaoqing Wang, Jason M. Millward, Shahriar Shalikar, Igor Fabian Tellez Ceja, Beate Endemann, Sonia Waiczies, Joseph Kuchling, Friedemann Paul, Georg Rose, Min-Chi Ku, Franz Schmitt, Thoralf Niendorf

**S1. Detailed k-space Trajectory Formulation**

The spoke angles for the three radial sampling schemes implemented in this work, uniform, rotated golden angle, and tiny golden angles, were calculated as follows.

*Uniform scheme.* The angle increment for the RARE module is set to ${\Delta\theta}_{RARE}=\pi/{n_{RARE}}$, where $n_{RARE}=N_{shot} \cdot{ETL}_{RARE}$, $N_{shot}$ is the number of RF excitations and ${ETL}_{RARE}$ the echo train length of the RARE module. The spoke angles are calculated as:

$$\theta_{k}=mod\left( {\Delta\theta}_{RARE}\cdot k,2\pi\right), k=0,1,\ldots, \left( n_{RARE}-1 \right) (1)$$

For the EPI module$,$ ${\Delta\theta}_{EPI}=\pi/{(N_{shot}\cdot\left( {ETL}_{EPI}+1 \right))}$ and the spoke angles are calculated as:

$$\theta_{l,n}=mod\left( \theta_{l}^{lastRARE}+n\cdot\left( \pi-{\Delta\theta}_{EPI} \right), 2\pi\right), l=1,2,\ldots,N_{shot}, n=1,2,\ldots,{ETL}_{EPI} (2)$$

where $\theta_{l}^{lastRARE}$ is the angle of the last RARE spoke for excitation index $l$.

*Rotated Golden Angle Scheme*. The k-space trajectory for the first RARE echo follows the conventional golden angle increment[^1^](#_ENREF_1) ${\Delta\theta}_{RARE}\approx111.25^{\circ}$; spoke angles are calculated using Eq. (1) for $k=0,1,\ldots,(N_{shot}-1)$. For subsequent RARE echoes, their k-space trajectories retain the same sampling pattern as the first echo but are rotated by[^2^](#_ENREF_2):

$\emptyset\left( n \right)=mod\left( \left( n-1 \right)\cdot\frac{\pi}{N_{shot}}\cdot\frac{\sqrt{5}-1}{2},\frac{\pi}{N_{shot}} \right), n=2,3,\ldots, {ETL}_{RARE} (3)$

For the EPI module ${\Delta\theta}_{EPI}=\pi/{(N_{shot}\cdot\left( {ETL}_{EPI}+1 \right))}$ and the EPI angles are calculated using Eq. (2).

*Tiny Golden Angles Scheme.* The angle increment for the RARE module is set to the 7th tiny golden angle[^3^](#_ENREF_3) ${\Delta\theta}_{RARE}\approx23.6^{\circ}$; spoke angles are calculated using Eq. (1). For the EPI module, we used the 17th tiny golden ${\Delta\theta}_{EPI}\approx10.2^{\circ}$ to prevent excessive blip gradient amplitudes while maintaining incoherent k-space coverage; spoke angles are calculated using Eq. (2).

**S2. Image Co-Registration Details**

Image co-registration was performed using contrast-weighted images. For model-based reconstruction, where no weighted images were reconstructed, a synthesized echo image generated from the estimated parameter maps was used.

For the human validation experiments, 2D rigid co-registration to the MSE space was performed using the imregtform function in MATLAB (The MathWorks, Inc., Natick, MA). The second-echo MSE image was used as the fixed image, while the first-echo MGRE image and the second-echo synthetic RARE image from 2in1-RARE-EPI were used as moving images. The resulting transformations were then applied to the corresponding quantitative maps using the MATLAB function imwarp with linear interpolation.

For the test-retest experiment, 2D rigid co-registration was performed in the same manner, with the second-echo synthetic RARE image of the test scan used as the fixed image, and the second-echo synthetic RARE image of the retest scan used as the moving image.

For the retrospective undersampling experiment, all undersampled model-based and PICS T_2_ maps were co-registered to the CG-SENSE T_2_ reference. The fixed image was the second-echo RARE image from the CG-SENSE reconstruction, while the second-echo RARE image from PICS and the second-echo synthetic RARE image from 2in-RARE-EPI used as the moving images. The same co-registration procedure was applied to the T_2_* maps.

**Supporting Information Figures**


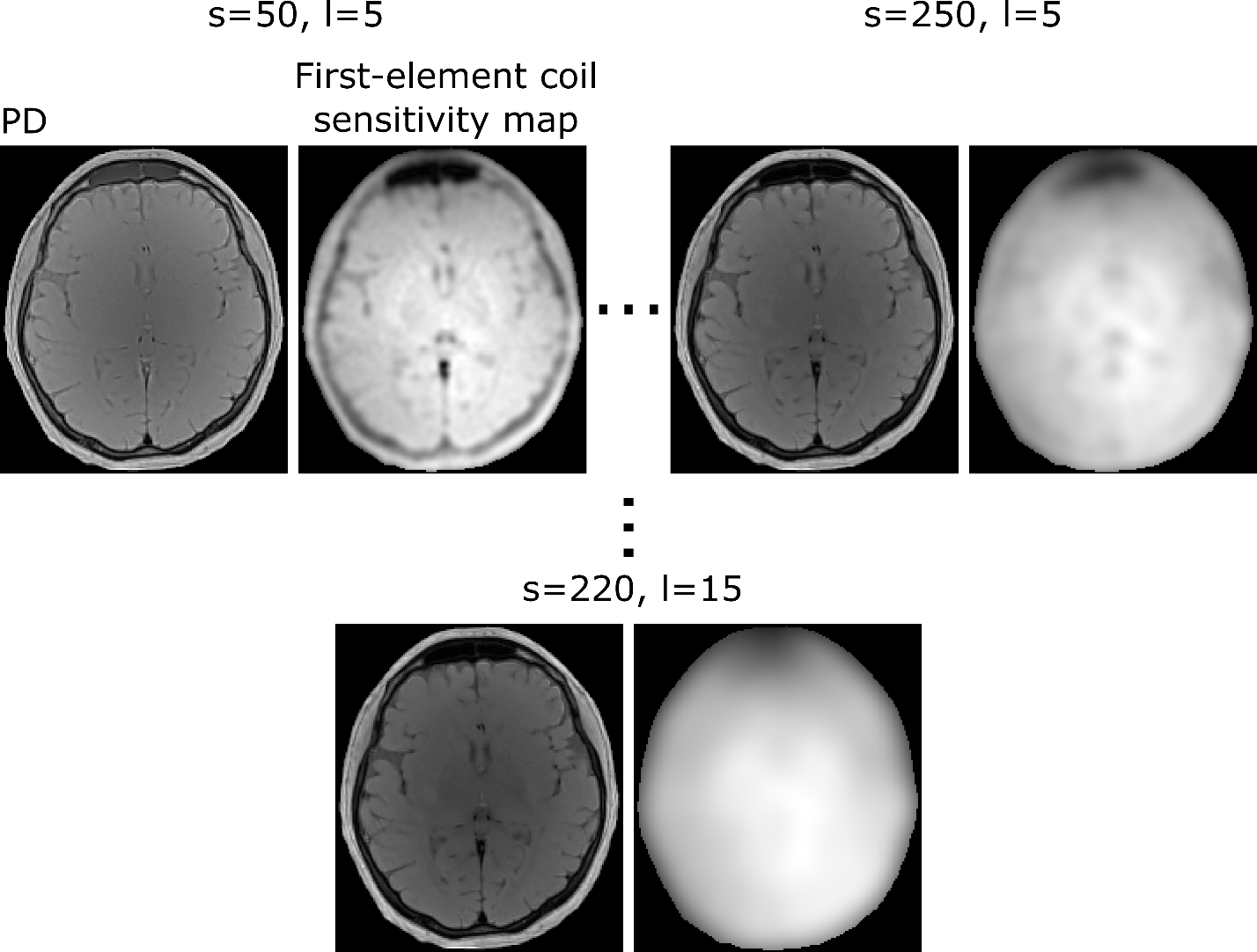


**Figure S1.** Model-based PD maps and magnitudes of the first-element coil sensitivity maps as a function of the Sobolev regularization parameters ($s, l$) for subject #2. When these values are too low, the coil sensitivity maps contain a large part of the image content as in the ($s=50, l=5$) case. The pair ($s=220, l=15$) produced smooth coil sensitivity maps and was selected for our study.


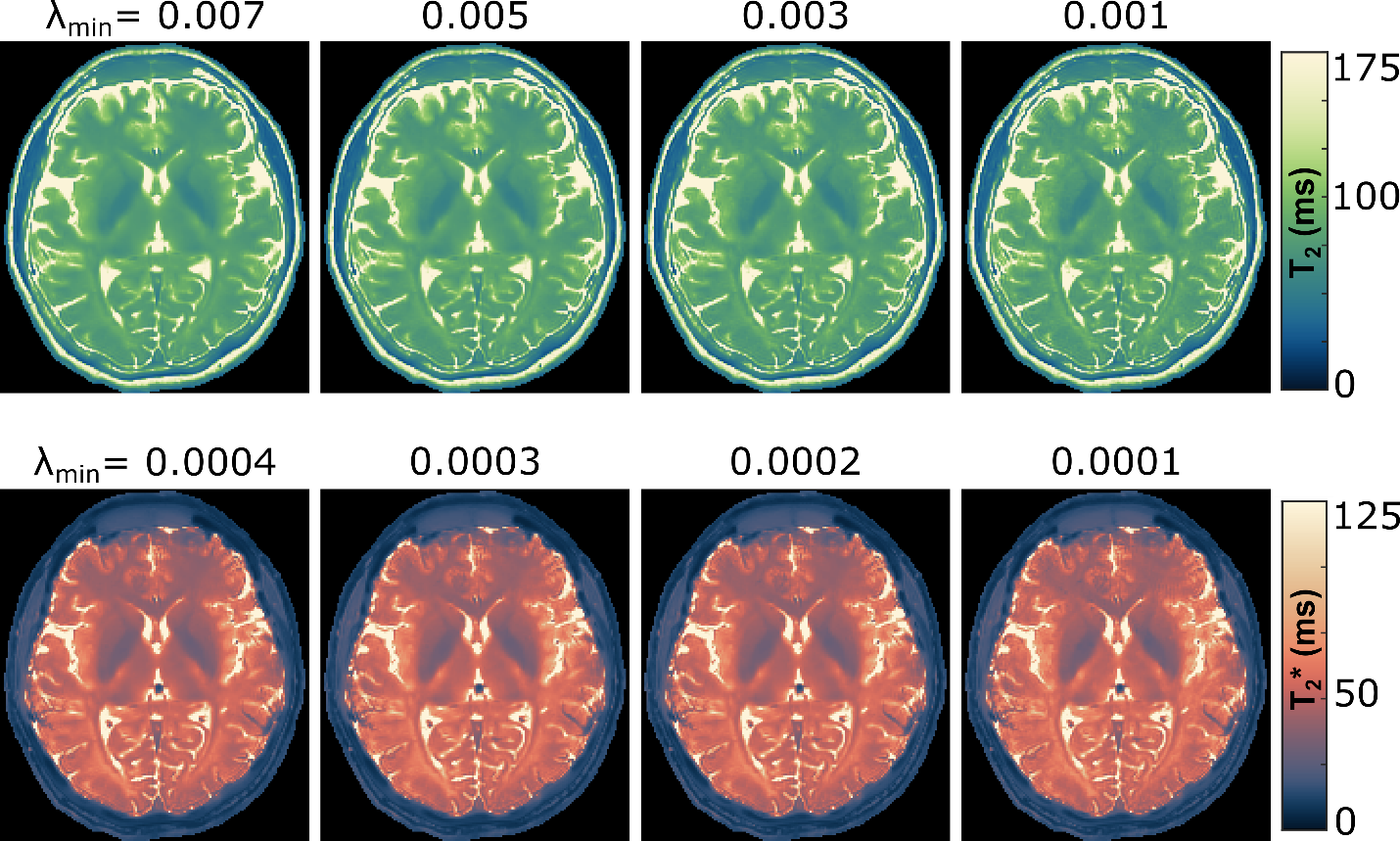


**Figure S2.** Model-based T_2_ and T_2_* maps as a function of the regularization parameter $\lambda_{min}$ for subject #2. A value of 0.005 was used for T_2_ mapping and 0.0002 for T_2_* mapping in our study.


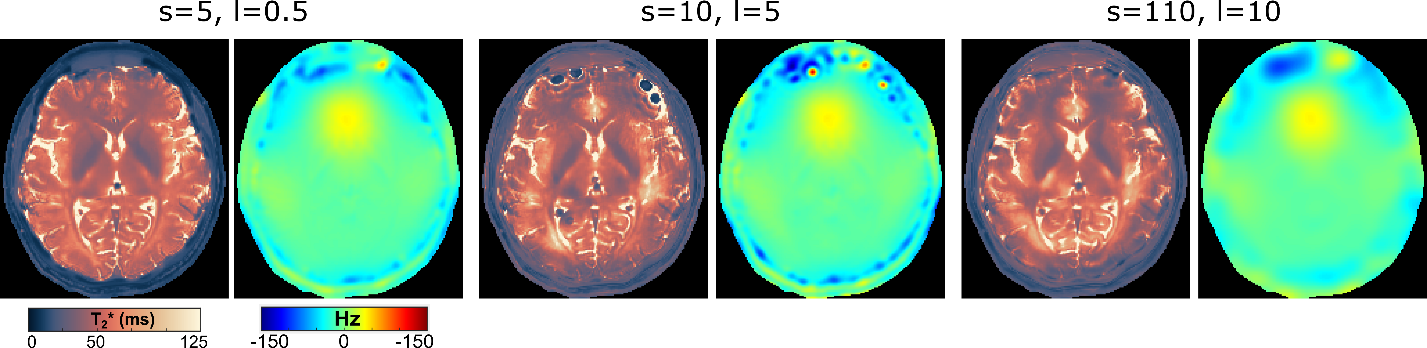


**Figure S3.** Model-based T_2_* and B_0_ maps as a function of the Sobolev regularization parameters ($s, l$) for subject #2. Similar to the coil sensitivity maps, the B_0_ map is assumed to be a smooth spatial function; however, it must also be able to capture local field variations, such as those near air/tissue interfaces. The parameter pair ($s=5, l=0.5$) produced smooth B_0_ maps that fulfill this requirement and was selected for our study.


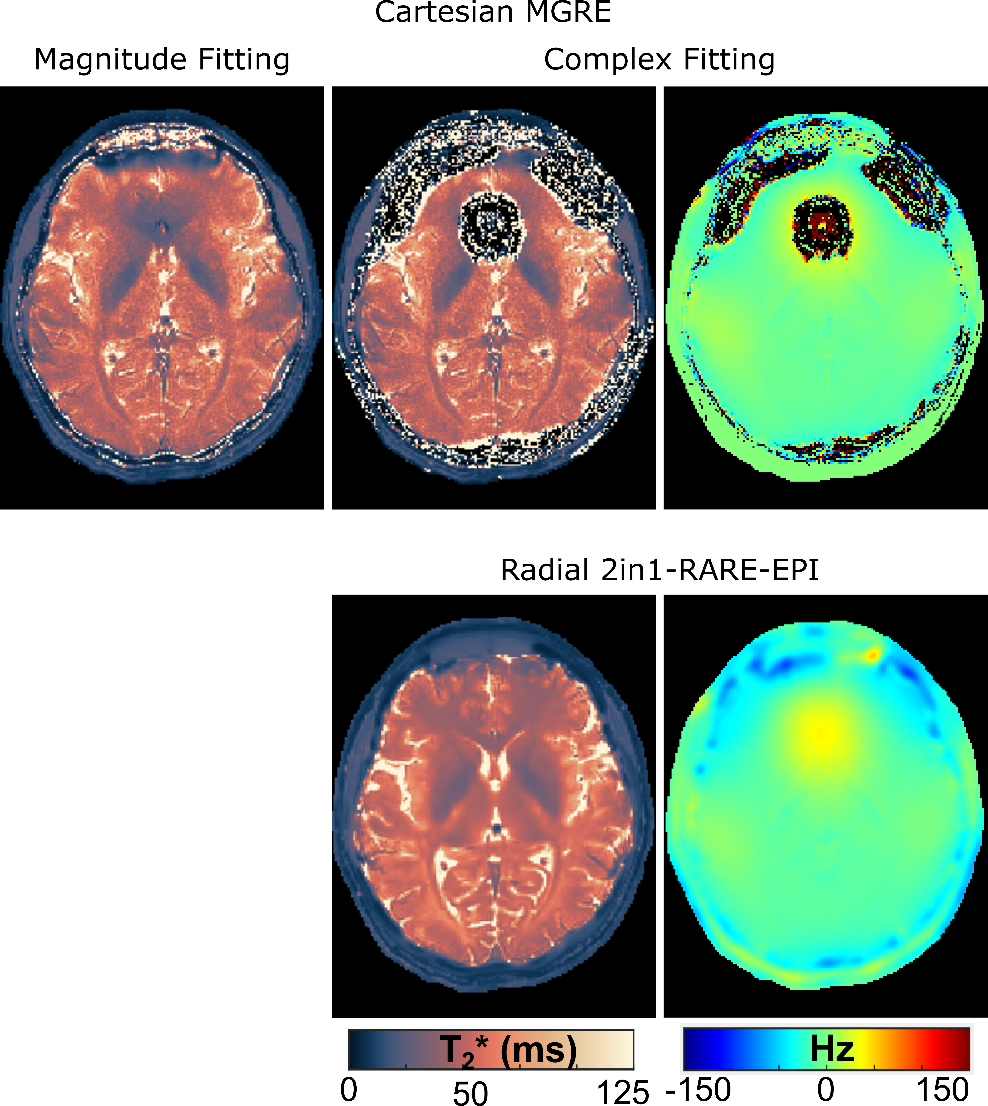


**Figure S4.** Top row: Results from voxel-wise fitting of the reference Cartesian MGRE data. Shown are the T₂* map obtained with magnitude fitting (left), the T₂* map obtained with complex fitting (middle), and the corresponding voxel-wise B₀ map (right). Complex fitting exhibits instabilities in regions with strong susceptibility gradients, leading to corrupted T₂* estimates and discontinuities in the B₀ map. Bottom row: T₂* and B₀ maps obtained from the accelerated 2in1-RARE-EPI acquisition using non-linear model-based reconstruction, which provides spatially smooth parameter estimates even in regions affected by large B₀ variations.


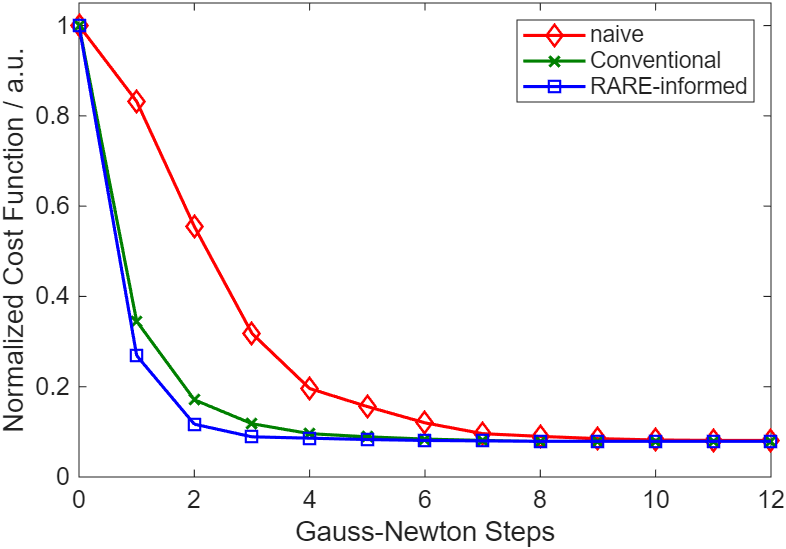


**Figure S5.** Normalized cost function as a function of Gauss-Newton steps for different initialization strategies for T_2_* mapping with non-linear model-based reconstruction of accelerated 2in1-RARE-EPI. In the naive initialization, $M_{0}^{'}=1, R_{2}^{*}=0, and f_{B_{0}}=0$. In the conventional initialization $M_{0}^{'}=0.1, R_{2}^{*}=0$, and $f_{B_{0}}$ was estimated from the first three EPI echoes[^4^](#_ENREF_4). In the RARE-informed initialization, $M_{0}^{'}$ was set to the last synthetic RARE echo image, $R_{2}^{*}=2\cdot R_{2}$, and $f_{B_{0}}$ was likewise estimated from the first three EPI echoes. The RARE-informed approach shows faster convergence than the naive and conventional strategies.


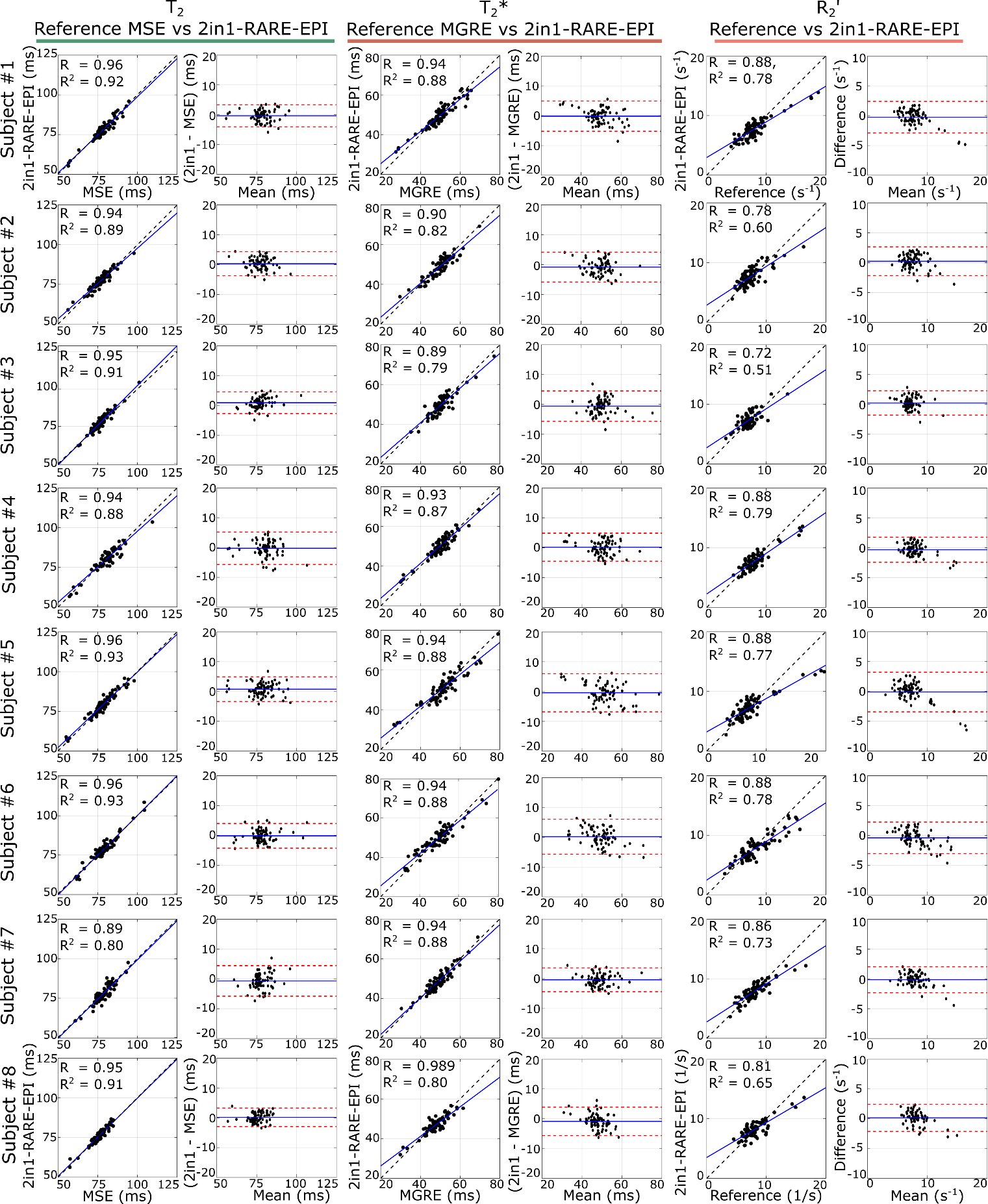


**Figure S6.** Scatter and Bland-Altman plots for eight healthy subjects. Statistical analysis of 77 ROIs per subject (7×7 pixels, 11 ROIs per slice) shows a strong correlation (scatter plots) and a small bias (Bland-Altman plots) between accelerated 2in1-RARE-EPI and the reference methods across all subjects for all T_2_ and T_2_*. For R_2_′ mapping, correlations were lower, largely due to R_2_′ overestimation in the globi pallidi by the MGRE reference. Four ROIs located in the left and right globus pallidus across two slices accounted for most of this overestimation.


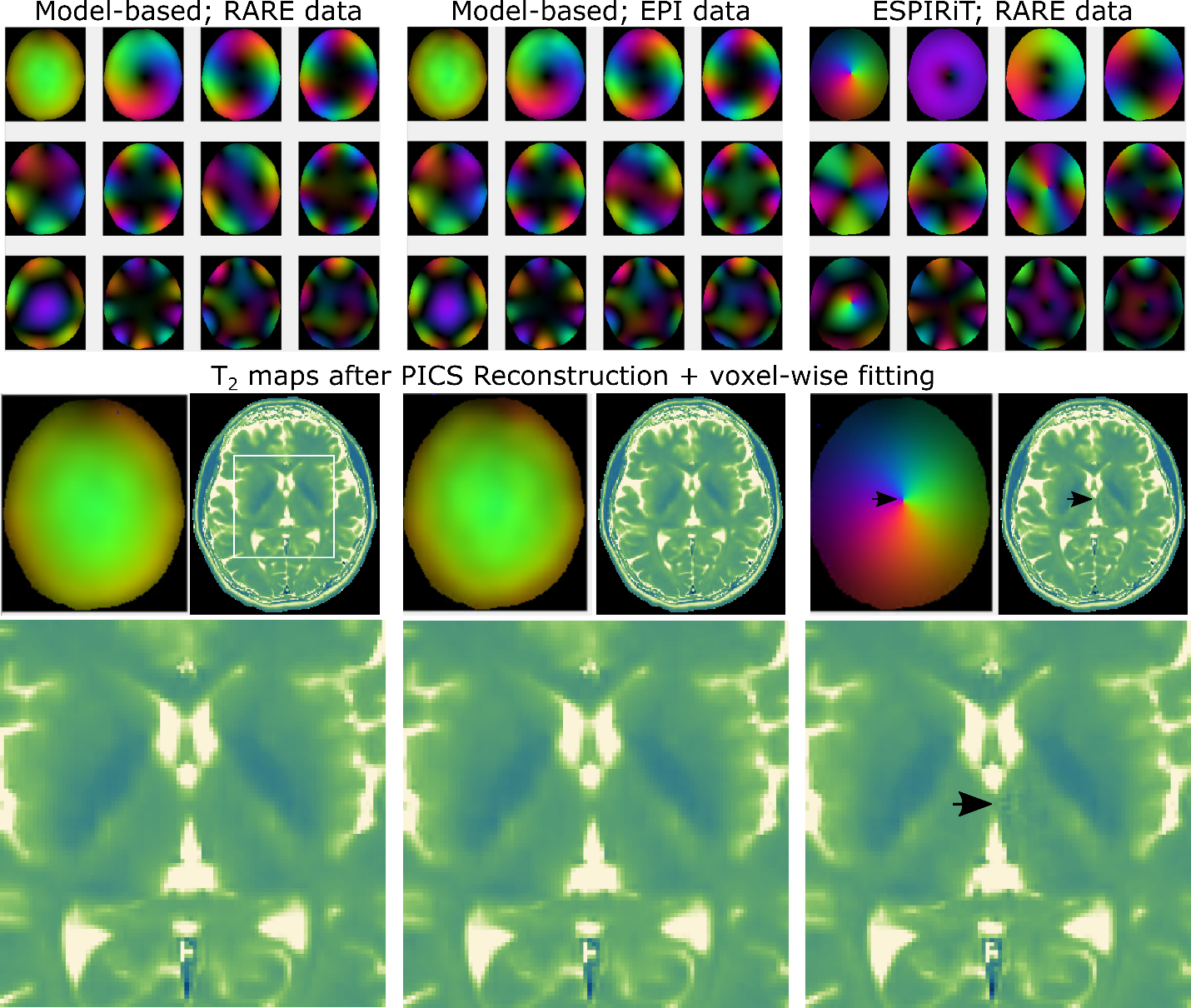


**Figure S7.** Comparison of coil sensitivity maps obtained with nonlinear model-based reconstruction and the ESPIRiT[^5^](#_ENREF_5) method. Top row: model-based coil sensitivities from the RARE and EPI module of 2in1-RARE-EPI data, and ESPIRiT sensitivities from the first echo of the RARE module. The three sets show similar spatial patterns, with ESPIRiT showing a phase pole in the principal (first) channel. Middle row: for each method above, the first sensitivity map is shown together with a T_2_ map obtained after voxel-wise fitting of echo images reconstructed using PICS with the respective coil sensitivities. T_2_ maps obtained with coil sensitivities from the non-linear model-based approaches appear similar, whereas the T_2_ map obtained with ESPIRiT sensitivities shows a localized artifact at the same position as the phase pole in the first channel. Bottom row: Magnified views of the T_2_ maps highlighting the artifact location.


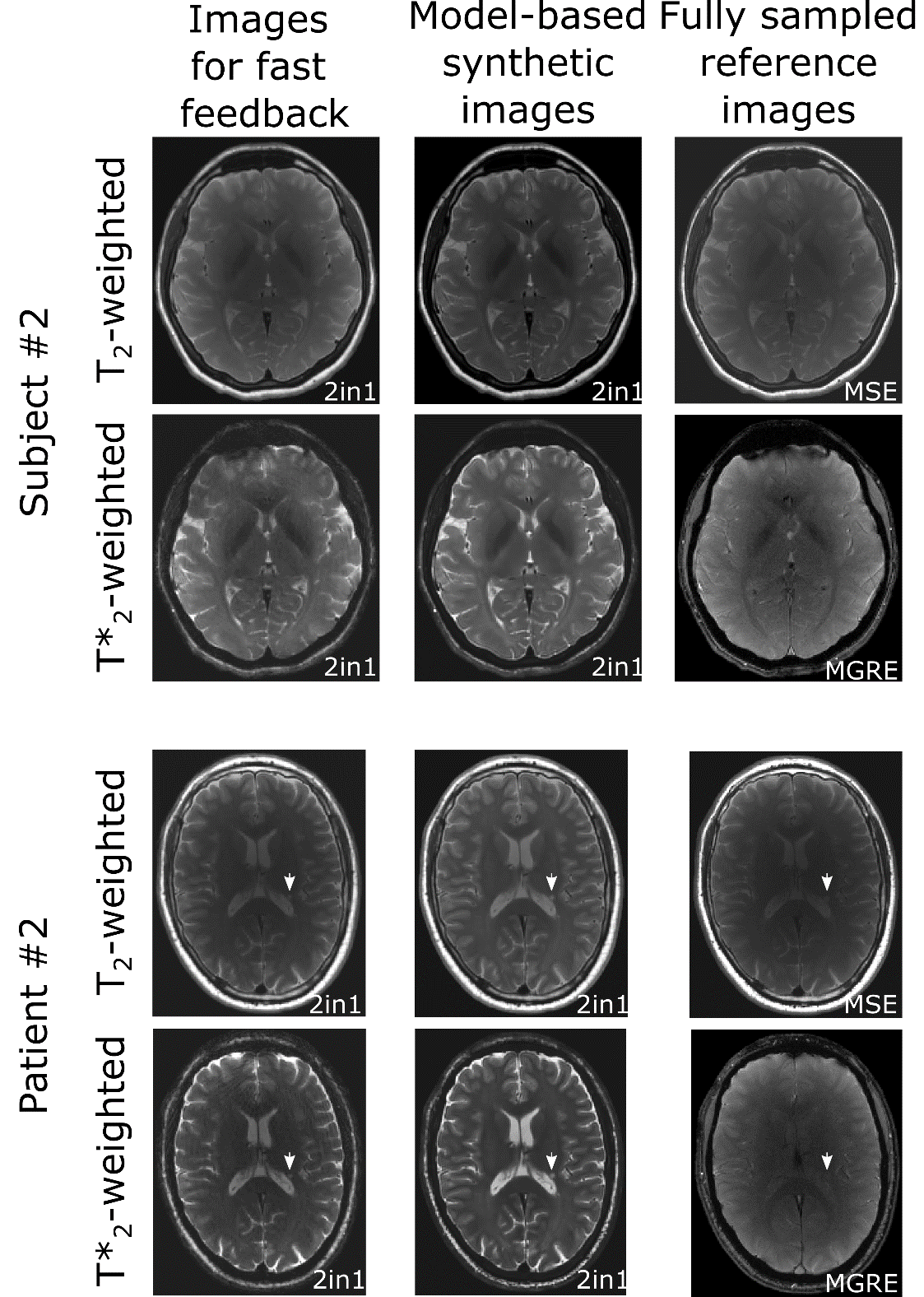


**Figure S8.** Weighted images reconstructed for fast feedback from the accelerated 2in1-RARE-EPI acquisition (left column), compared with non-linear model-based synthetic images (middle column) and fully sampled Cartesian reference images (right column). The fast-feedback images are obtained from 228 radial spokes corresponding to echoes 9-14 of the RARE module and echoes 7-12 of the EPI module. Reconstruction of the fast-feedback images requires less than 4s using PICS reconstruction. In contrast, generating the model-based synthetic images requires full estimation of the quantitative parameter maps, which takes approximately 132s per slice. White arrows indicate a MS lesion in patient #2, which is visible in all weighted images, although it is more difficult to visualize in the MGRE T2*-weighted reference image.


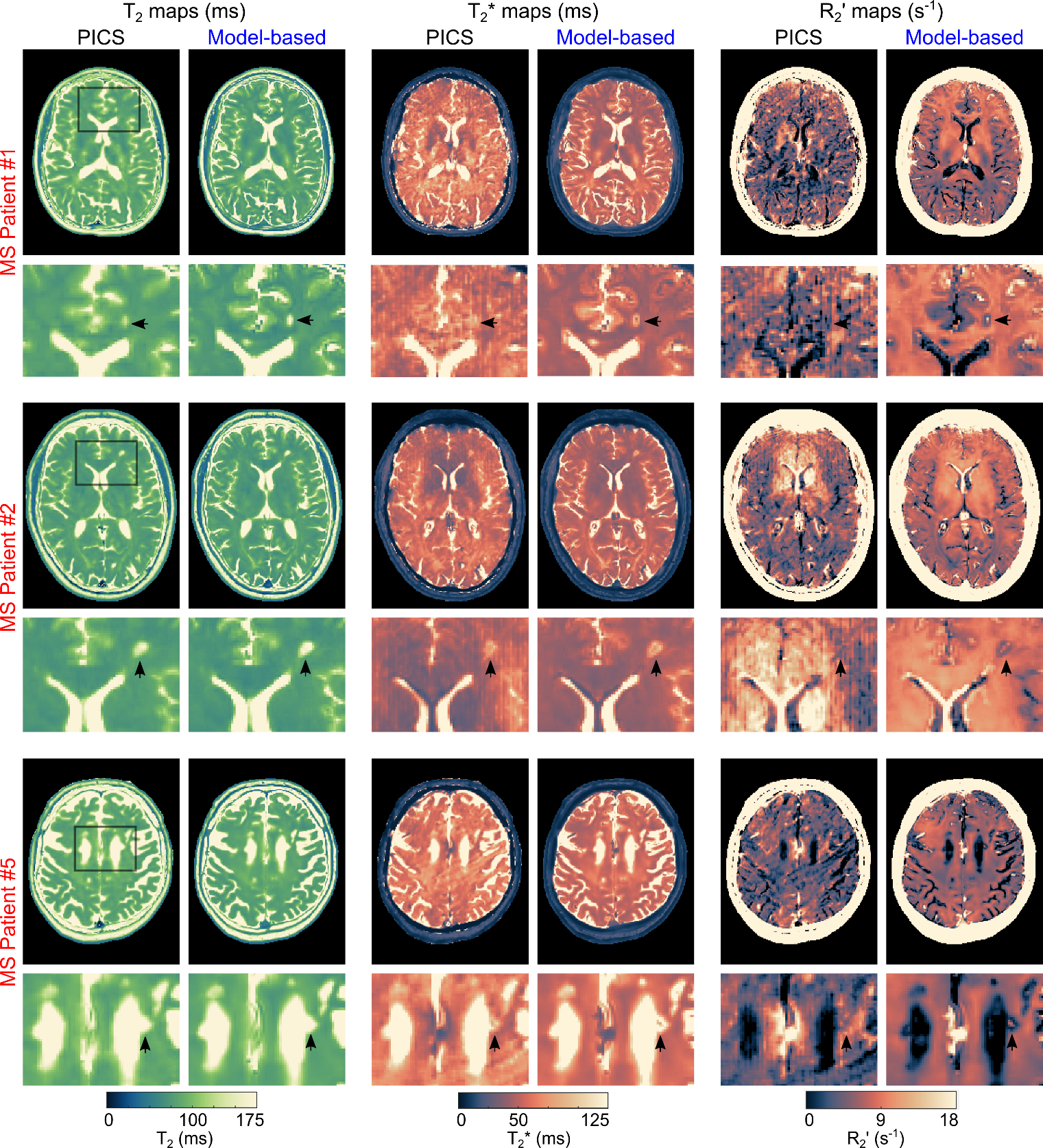


**Figure S9.** Comparison of PICS and nonlinear model-based reconstruction of 2in1-RARE-EPI data in MS patients. T_2_ maps obtained with both PICS and model-based reconstruction of the same accelerated 2in-RARE-EPI data show comparable depiction of focal lesions (arrows) in three representative MS patients. However, the model-based reconstruction enables clear visualization of intralesional veins on the corresponding T_2_* and R_2_′ maps, appearing as a centrally located high-susceptibility structure within the lesions. In contrast, these features are difficult to visualize in the T_2_* and R_2_′ maps using PICS reconstruction.

**Supporting Information Videos**

**Supporting Information Videos S1-S8:** $T_{2}, T_{2}^{*}, and R_{2}^{'}$ maps obtained with 2in1-RARE-EPI and nonlinear model-based reconstruction, and with the reference MSE and MGRE, together with absolute error maps relative to the references and the corresponding ROI locations, for subjects 1-8. Model-based B_0_ maps are also shown in the videos.

**Supporting Information Videos S9-S10:** $PD, T_{2}, T_{2}^{*}, and R_{2}^{'}$ maps obtained with 2in1-RARE-EPI and nonlinear model-based reconstruction in a test-retest experiment, for two subjects, as well as absolute error maps relative to the test scan, and the corresponding ROI locations, across all 27 slices.

**References**

1. Winkelmann S, Schaeffter T, Koehler T, Eggers H, Doessel O. An optimal radial profile order based on the Golden Ratio for time-resolved MRI. *IEEE transactions on medical imaging*. 2006;26(1):68-76. <https://doi.org/10.1109/tmi.2006.885337>

2. Zhou Z, Han F, Yan L, Wang DJ, Hu P. Golden‐ratio rotated stack‐of‐stars acquisition for improved volumetric MRI. *Magnetic resonance in medicine*. 2017;78(6):2290-2298. <https://doi.org/10.1002/mrm.26625>

3. Wundrak S, Paul J, Ulrici J, Hell E, Rasche V. A small surrogate for the golden angle in time-resolved radial MRI based on generalized fibonacci sequences. *IEEE transactions on medical imaging*. 2014;34(6):1262-1269. <https://doi.org/10.1109/tmi.2014.2382572>

4. Tan Z, Voit D, Kollmeier JM, Uecker M, Frahm J. Dynamic water/fat separation and inhomogeneity mapping—joint estimation using undersampled triple‐echo multi‐spoke radial FLASH. *Magnetic Resonance in Medicine*. 2019;82(3):1000-1011. <https://doi.org/10.1002/mrm.27795>

5. Uecker M, Lai P, Murphy MJ, et al. ESPIRiT—an eigenvalue approach to autocalibrating parallel MRI: where SENSE meets GRAPPA. *Magnetic resonance in medicine*. 2014;71(3):990-1001. <https://doi.org/10.1002/mrm.24751>
