# Supplementary figures and images for: Simultaneous T 2, T 2*, and R 2′ Mapping for Multiple Sclerosis Using Nonlinear Model‐Based Reconstruction of Undersampled Radial RARE‐EPI MRI
Source: Magn Reson Med. 2026 Jun 18;96(4):1872–92. doi: 10.1002/mrm.70465 (PMC13419268; doi:10.1002/mrm.70465)

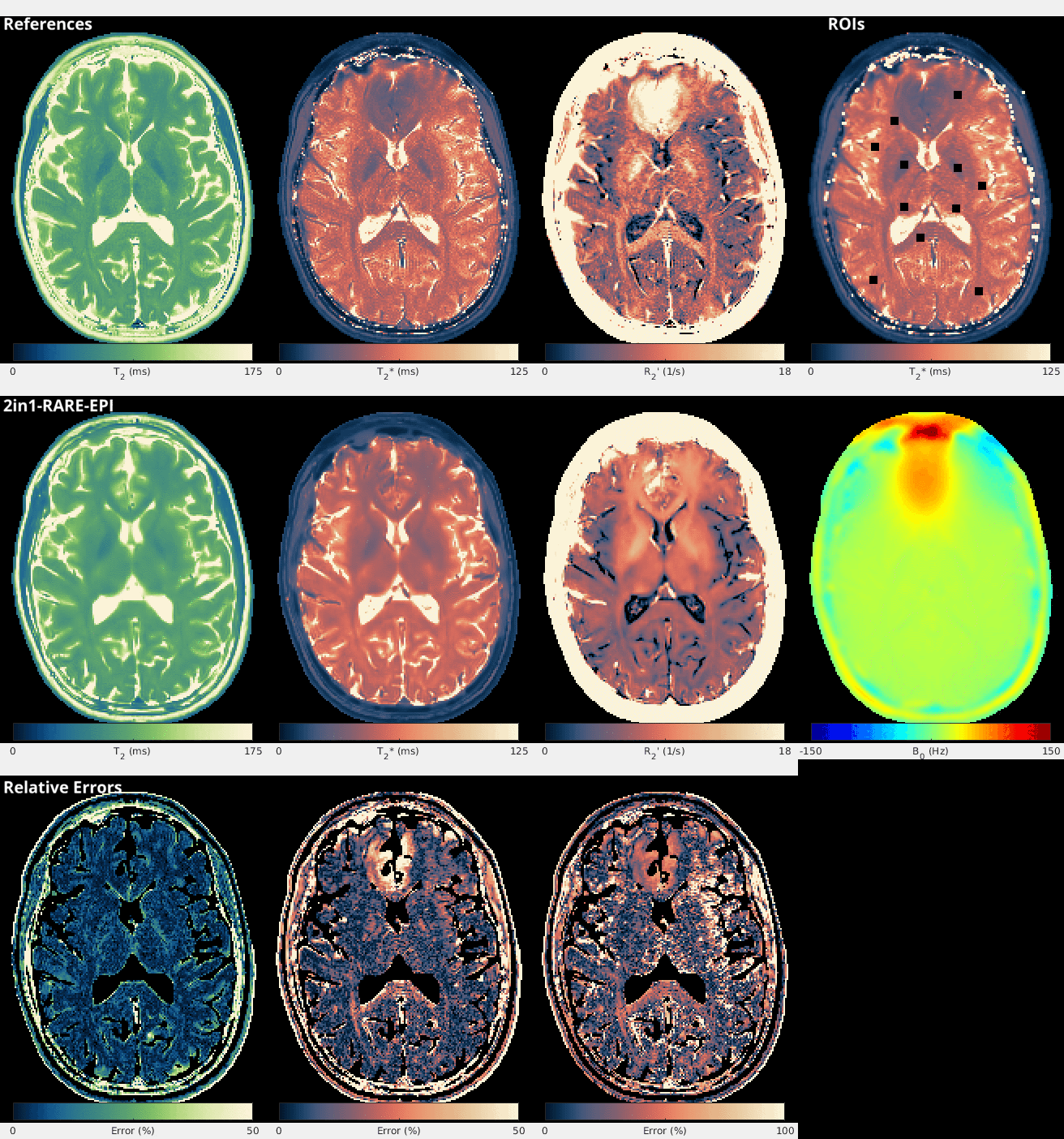

Supplement: Supplementary file 2 — Videos S1–S8: mrm70465‐sup‐0002‐Video_S1‐S8.zip. T2,T2*,andR2′ maps obtained with 2in1‐RARE‐EPI and nonlinear model‐based reconstruction, and with the reference MSE and MGRE, together with absolute error maps relative to the references and the corresponding ROI locations, for subjects 1–8. [file MRM-96-1872-s003.zip › Video S2.gif]

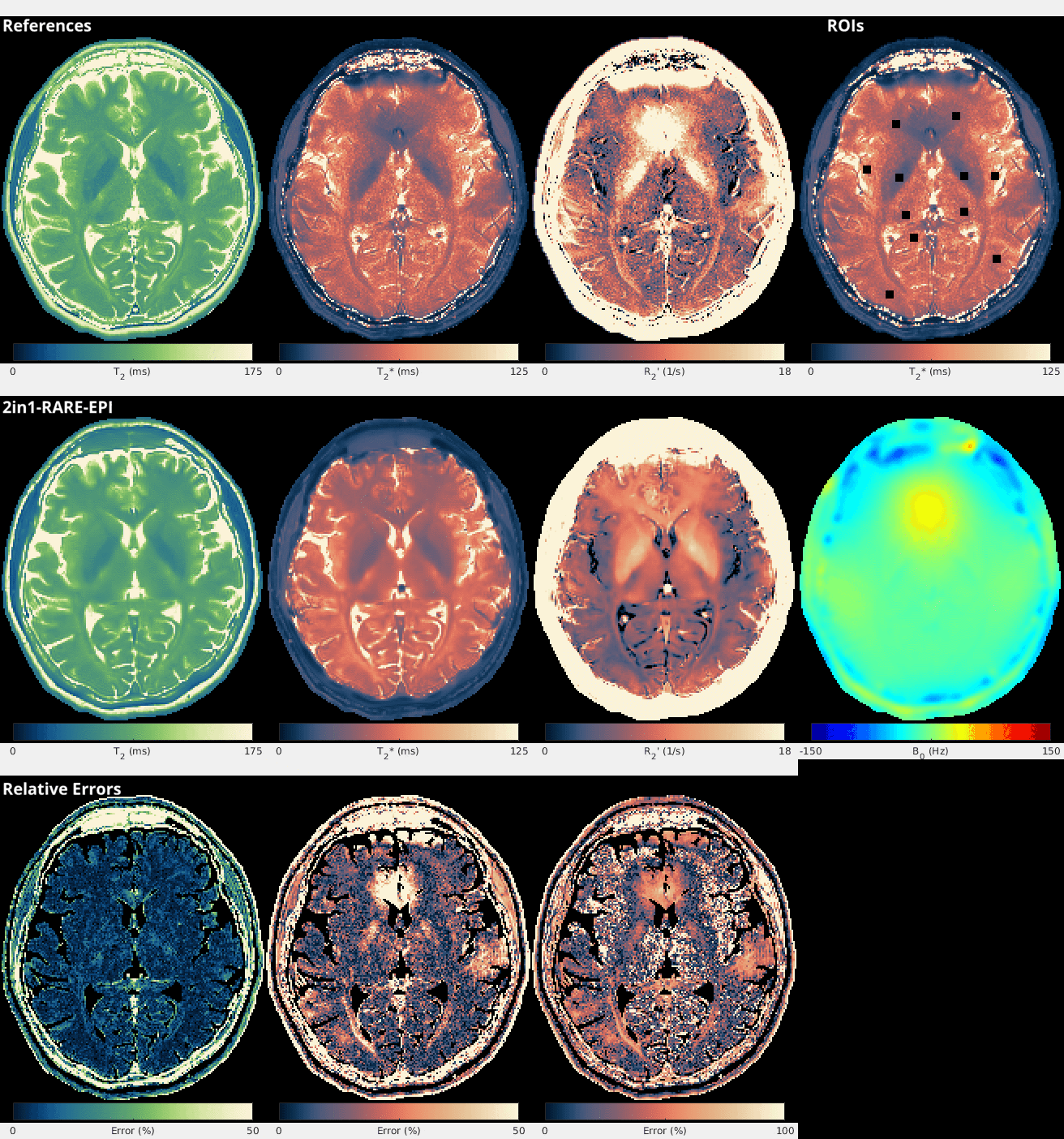

Supplement: Supplementary file 2 — Videos S1–S8: mrm70465‐sup‐0002‐Video_S1‐S8.zip. T2,T2*,andR2′ maps obtained with 2in1‐RARE‐EPI and nonlinear model‐based reconstruction, and with the reference MSE and MGRE, together with absolute error maps relative to the references and the corresponding ROI locations, for subjects 1–8. [file MRM-96-1872-s003.zip › Video S5.gif]

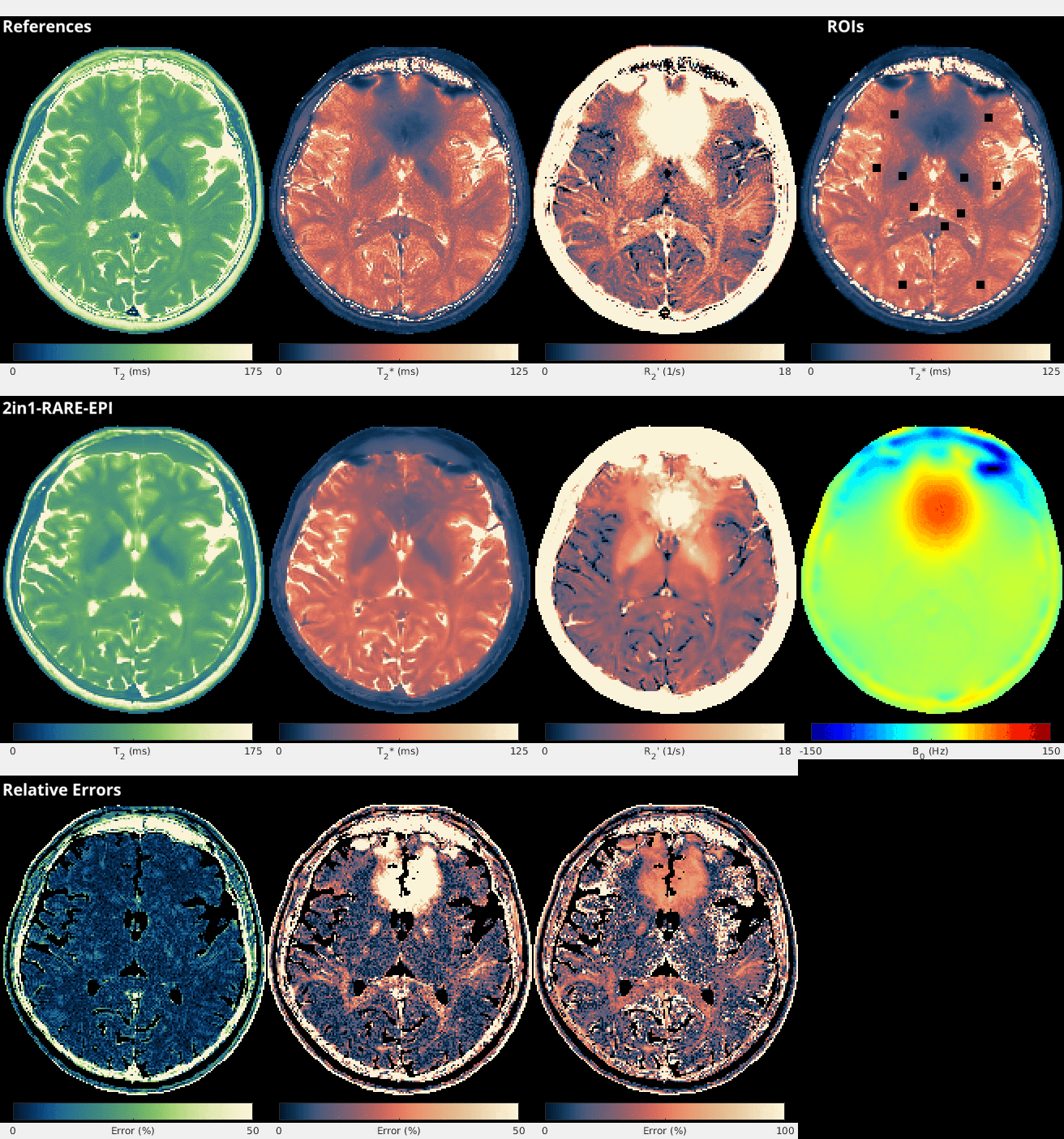

Supplement: Supplementary file 2 — Videos S1–S8: mrm70465‐sup‐0002‐Video_S1‐S8.zip. T2,T2*,andR2′ maps obtained with 2in1‐RARE‐EPI and nonlinear model‐based reconstruction, and with the reference MSE and MGRE, together with absolute error maps relative to the references and the corresponding ROI locations, for subjects 1–8. [file MRM-96-1872-s003.zip › Video S1.gif]

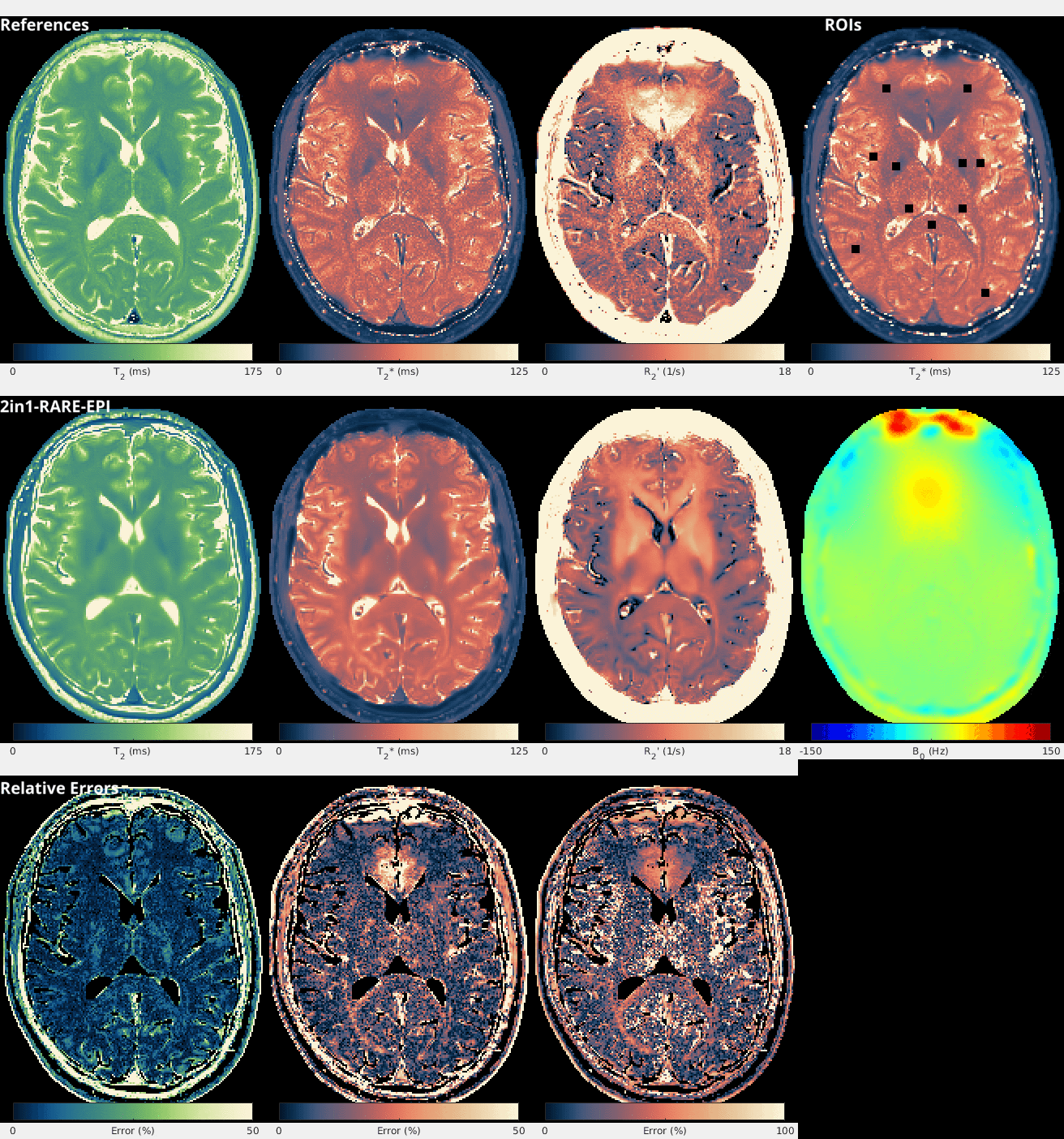

Supplement: Supplementary file 2 — Videos S1–S8: mrm70465‐sup‐0002‐Video_S1‐S8.zip. T2,T2*,andR2′ maps obtained with 2in1‐RARE‐EPI and nonlinear model‐based reconstruction, and with the reference MSE and MGRE, together with absolute error maps relative to the references and the corresponding ROI locations, for subjects 1–8. [file MRM-96-1872-s003.zip › Video S3.gif]

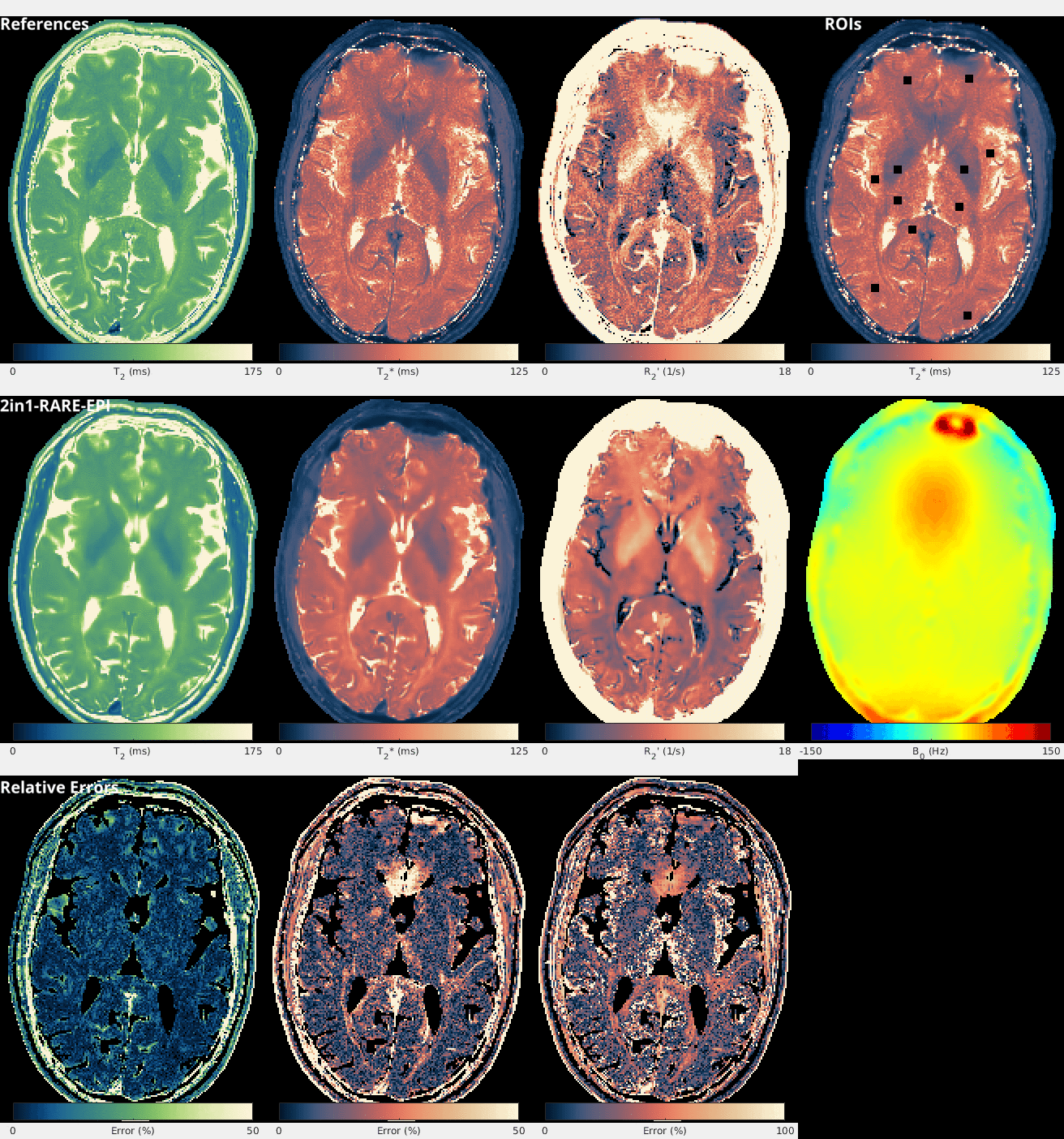

Supplement: Supplementary file 2 — Videos S1–S8: mrm70465‐sup‐0002‐Video_S1‐S8.zip. T2,T2*,andR2′ maps obtained with 2in1‐RARE‐EPI and nonlinear model‐based reconstruction, and with the reference MSE and MGRE, together with absolute error maps relative to the references and the corresponding ROI locations, for subjects 1–8. [file MRM-96-1872-s003.zip › Video S4.gif]

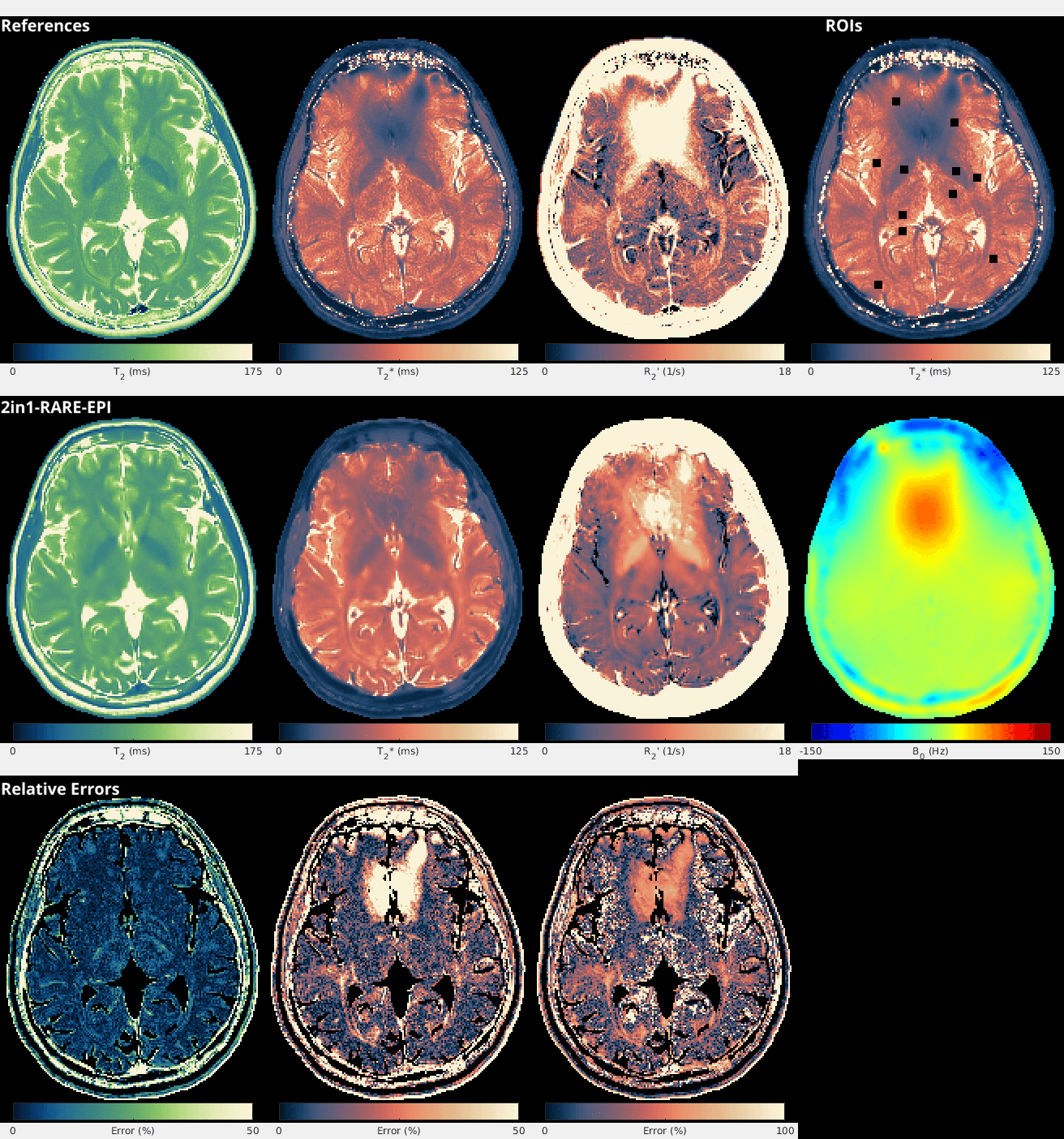

Supplement: Supplementary file 2 — Videos S1–S8: mrm70465‐sup‐0002‐Video_S1‐S8.zip. T2,T2*,andR2′ maps obtained with 2in1‐RARE‐EPI and nonlinear model‐based reconstruction, and with the reference MSE and MGRE, together with absolute error maps relative to the references and the corresponding ROI locations, for subjects 1–8. [file MRM-96-1872-s003.zip › Video S6.gif]

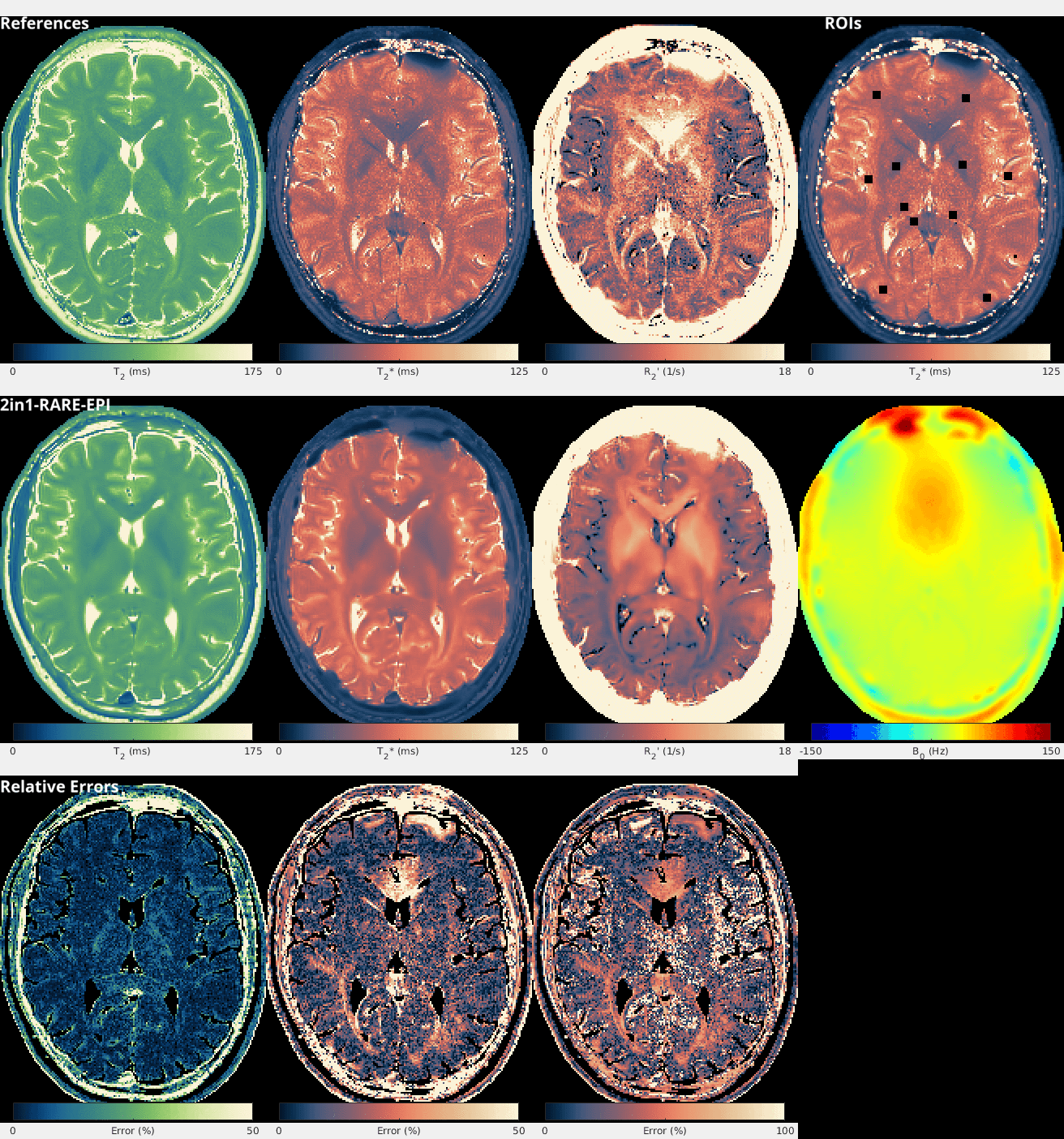

Supplement: Supplementary file 2 — Videos S1–S8: mrm70465‐sup‐0002‐Video_S1‐S8.zip. T2,T2*,andR2′ maps obtained with 2in1‐RARE‐EPI and nonlinear model‐based reconstruction, and with the reference MSE and MGRE, together with absolute error maps relative to the references and the corresponding ROI locations, for subjects 1–8. [file MRM-96-1872-s003.zip › Video S7.gif]

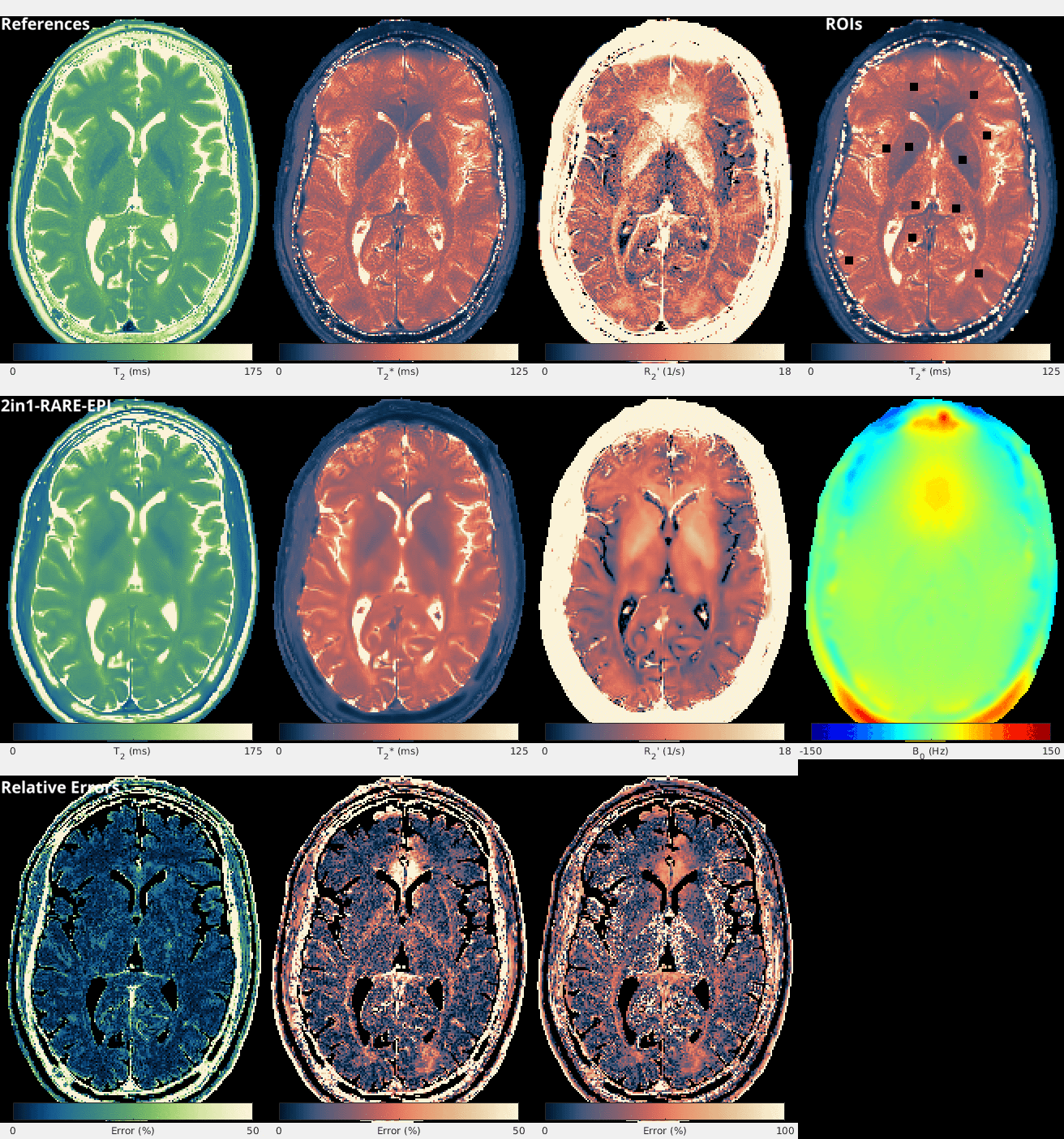

Supplement: Supplementary file 2 — Videos S1–S8: mrm70465‐sup‐0002‐Video_S1‐S8.zip. T2,T2*,andR2′ maps obtained with 2in1‐RARE‐EPI and nonlinear model‐based reconstruction, and with the reference MSE and MGRE, together with absolute error maps relative to the references and the corresponding ROI locations, for subjects 1–8. [file MRM-96-1872-s003.zip › Video S8.gif]

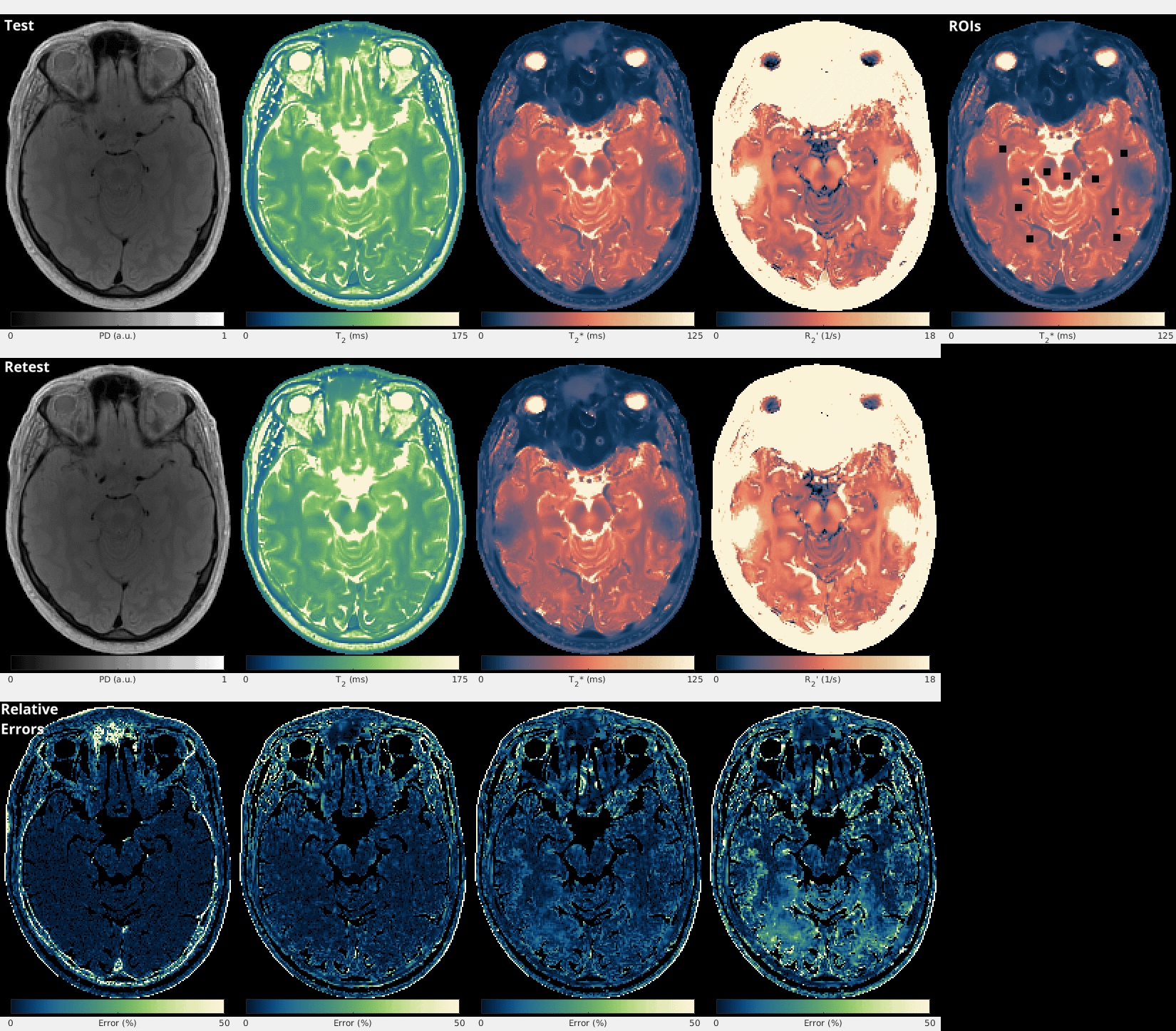

Supplement: Supplementary file 3 — Videos S9 and S10: PD,T2,T2*,andR2′ maps obtained with 2in1‐RARE‐EPI and nonlinear model‐based reconstruction in a test–retest experiment, for two subjects, as well as absolute error maps relative to the test scan, and the corresponding ROI locations, across all 27 slices. [file MRM-96-1872-s001.zip › Video S9.gif]

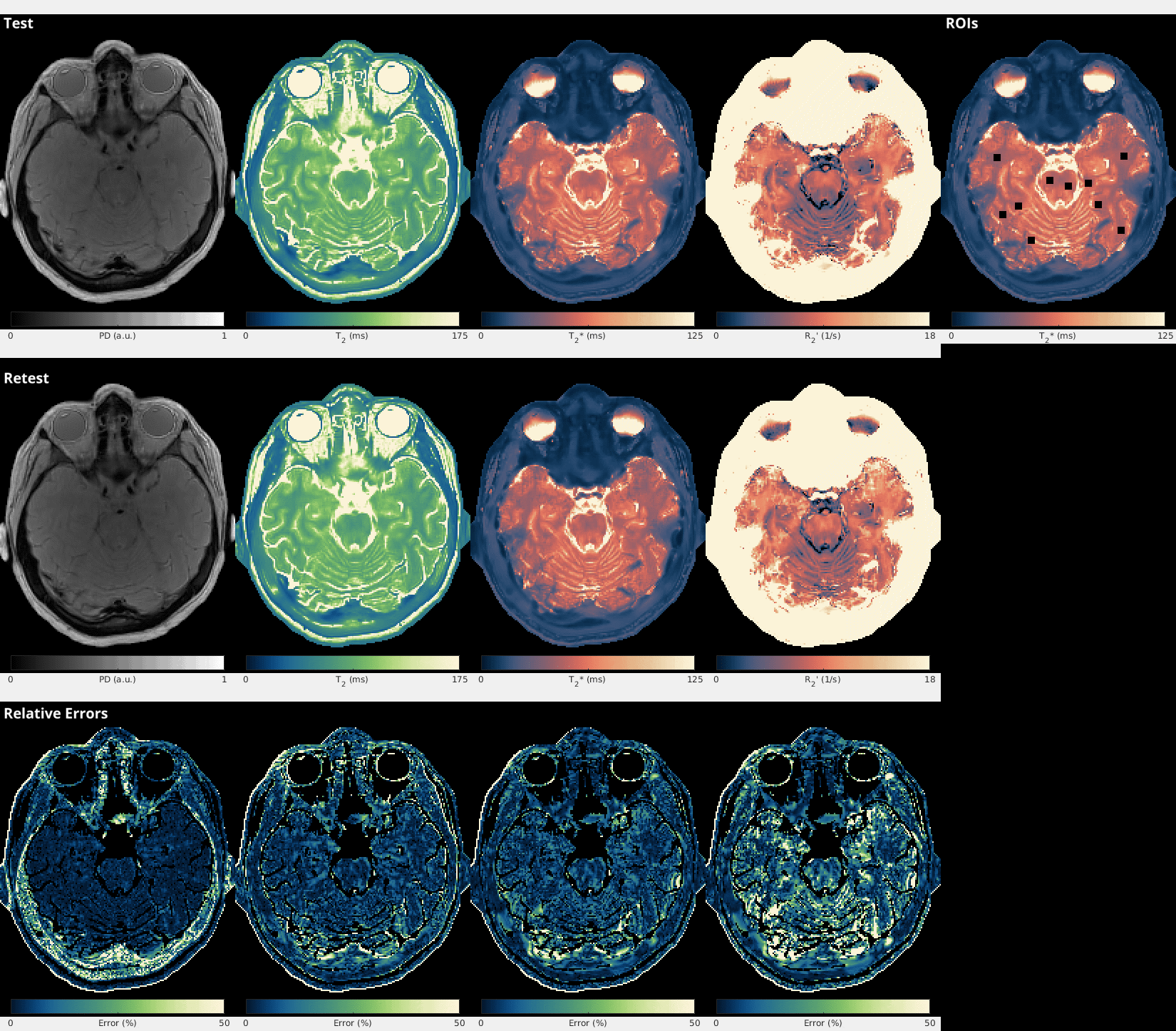

Supplement: Supplementary file 3 — Videos S9 and S10: PD,T2,T2*,andR2′ maps obtained with 2in1‐RARE‐EPI and nonlinear model‐based reconstruction in a test–retest experiment, for two subjects, as well as absolute error maps relative to the test scan, and the corresponding ROI locations, across all 27 slices. [file MRM-96-1872-s001.zip › Video S10.gif]
